# Multiomics Approach Reveals the Inhibitory Effects of Protocatechuic Acid on the Marine Dinoflagellate *Scrippsiella acuminata*

**DOI:** 10.3390/microorganisms14030561

**Published:** 2026-03-01

**Authors:** Xin Zhang, Meiyao He, Di Wang, Meimei Wang, Hongxin Liu, Jihui Wang, Shunshan Duan, Meng Liu

**Affiliations:** 1 School of Life and Health Technology, Dongguan University of Technology, Dongguan 523808, China; 2 Department of Ecology, Jinan University, Guangzhou 510632, China

**Keywords:** *Scrippsiella acuminata*, protocatechuic acid, multiomics analysis, allelopathic stress, harmful algae blooms

## Abstract

Harmful algal blooms have occurred more frequently in recent decades and threaten aquaculture, tourism and human health. As a promising control method, most studies on allelopathic mechanisms have focused on the physiological effects on harmful algae. This study employed a multiomics approach to investigate the allelopathic response of the dinoflagellate *Scrippsiella acuminata* to the allelochemical protocatechuic acid, a phenolic compound known for its inhibitory effects on algal growth. Using transcriptomic, proteomic, and metabolomic analyses, we identified significant changes in gene expression (5247 upregulated and 81 downregulated), protein expression (56 upregulated and 49 downregulated), and metabolite profiles (320 upregulated and 168 downregulated) in response to allelochemical stress. Transcriptomic data revealed an upregulation of genes associated with antioxidant systems and energy metabolism, suggesting a potential antioxidant response to protocatechuic acid exposure. Proteomic analysis highlighted the impact on photosynthesis, energy metabolism, and genetic information processing, with a particular emphasis on the modulation of lipid and carbohydrate metabolism to adapt to stress. Metabolomic profiling corroborated these findings, demonstrating shifts in lipid and amino acid metabolism indicative of an adaptive strategy for energy storage and maintenance of cellular homeostasis under allelochemical stress. Notably, alterations in photosynthesis-related proteins and metabolites indicated a direct effect of protocatechuic acid on the photosynthetic machinery, potentially impairing algal growth and energy production. In conclusion, our multiomics analysis provides a comprehensive view of the complex response of *S. acuminata* to allelochemical stress, revealing the intricate interplay among genetic, proteomic, and metabolic adjustments. These insights contribute to the understanding of allelopathic interactions and offer potential avenues for the development of novel strategies to manage harmful algal blooms.

## 1. Introduction

Red tides are among the most well-known phenomena associated with harmful algal blooms (HABs) and are capable of discoloring water bodies. The occurrence of red tides can have severe impacts on coastal and aquatic ecosystems [1]. Common harmful algae include *Alexandrium*, *Dinophysis*, *Gymnodinium*, *Noctiluca*, *Scrippsiella* and *Karenia* species [2]. HABs can occur repeatedly, and each occurrence can exert significant negative effects on coastal aquaculture, tourism, and human health, especially for HABs caused by dinoflagellates [3,4,5]. Due to the difficulty of HAB eradication and the continuous increase in human activities over the past few decades, the range and severity of HABs have gradually expanded [6].

The establishment of HAB control methods requires a combination of multidisciplinary approaches and strategies adapted to local conditions. Methods for controlling HABs primarily aim to reduce the population of toxin-producing microalgae and remove toxins from water, thereby limiting the impact of HABs. These control methods are based on physical, chemical, or biological principles and have been applied in both freshwater and marine ecosystems [7,8]. However, these methods often have drawbacks such as high costs, potential for secondary environmental pollution, threats to other aquatic life, and disruption of the ecosystem balance. In addition to harmful algal bloom species, model organisms such as the green alga *Chlamydomonas* reinhardtii have provided foundational insights into stress acclimation mechanisms. Comprehensive studies have detailed its adaptive responses to a wide range of environmental stresses, including oxidative stress [9], high light [10], and heavy metal exposure [11]. Recent advances, particularly the application of multiomics approaches, have enabled a systems-level understanding of these processes. For instance, a combined transcriptomic and phosphoproteomic analysis has elucidated the conserved and divergent pathways in osmotic stress signaling, highlighting the role of diverse cellular compartments [12]. Similarly, metabolomic profiling under nutrient deprivation has revealed the critical function of signaling molecules like inositol polyphosphates in regulating photosynthesis, cell division, and amino acid metabolism during stress adaptation [13]. Furthermore, studies on polyamine dynamics have integrated redox balance with amino acid pathways to explain growth regulation under stress [14]. These model systems offer a robust comparative framework for understanding how non-model harmful algae like *Scrippsiella acuminata* respond to environmental stressors, including allelochemicals

The discovery of allelopathy has provided a new, safe, efficient, and economical method for controlling HABs. Allelopathy usually refers to the process whereby an organism releases one or more chemical substances into the environment during its growth, which ultimately has a direct or indirect adverse effect on the growth, survival, and reproduction of other organisms [15]. These chemical substances are known as allelochemicals. Compared with traditional algicides, allelochemicals can be biodegraded and thus have the potential to cause less persistent environmental pollution. The biological phenomenon of allelopathy was initially discovered in terrestrial plants and subsequently gained attention for its role in aquatic plants. In recent years, research on the allelopathic effects of plants on microalgae has gradually increased [16]. However, it is important to acknowledge that allelochemicals are biologically active molecules that may exert non-target effects on co-occurring phytoplankton, bacteria, zooplankton, or other aquatic organisms [17,18,19]. The ecological consequences of introducing allelochemicals—even naturally derived ones—into complex aquatic ecosystems warrant careful consideration, as shifts in microbial community structure or disruption of food web dynamics could occur. Therefore, while allelopathy offers a promising strategy for HAB management, its application should be evaluated within a broader ecological risk assessment framework.

As more allelochemicals are isolated and identified, the mechanisms by which they inhibit algal growth are gradually being elucidated. Overall, the mechanisms by which allelochemicals inhibit algal growth include the following aspects: the effect of allelopathy on photosynthesis, the impact of allelopathy on cell structure, the influence of allelopathy on algal cell enzyme activity, and other physiological effects [16]. For instance, phenolic acids (e.g., p-coumaric acid) have been shown to suppress photosynthetic protein expression in cyanobacteria [20], while fatty acids released by aquatic plants can disrupt algal cell membrane integrity [21]. Common allelochemicals often include phenolic acids, fatty acids, tannic acids, lactones, terpenes, alkaloids, flavonoids, and sulfides [21,22,23,24,25]. The protocatechuic acid (PA) is a naturally occurring phenolic acid widely distributed in plants, including fruits, vegetables, and medicinal herbs [26]. It can be released directly into the environment, for example, from sweet sorghum (*Sorghum bicolor*) [27] or generated via the microbial degradation of larger phenolic compounds, such as the conversion of (-)-catechin from *Rhododendron formosanum* by soil *Pseudomonas* bacteria [28]. Thus, PA is an ecologically relevant allelochemical with both direct plant and indirect microbial origins. However, most of these studies have focused on cyanobacteria or diatoms, and there is relatively little research on the mechanisms of action of allelochemicals on dinoflagellates, especially at the molecular level. For example, while a linoleic acid has been reported to alter metabolite profiles in *Karenia mikimotoi* [29], the regulatory networks linking allelochemical exposure to dinoflagellate growth inhibition remain poorly characterized. The dinoflagellate *Scrippsiella acuminata* was selected as the target organism for several reasons that underscore its significance as a harmful algal bloom (HAB)-forming species. First, *S. acuminata* is a globally distributed and recurrent bloom-forming species, frequently responsible for red tides in coastal waters across Asia, Europe, and the Americas [30,31,32,33,34]. These blooms cause significant ecological and economic impacts, including water discoloration, oxygen depletion, and mass mortality of shellfish larvae [33]. Second, its ability to form resting cysts—a key survival strategy that facilitates bloom recurrence and geographic expansion—makes it particularly challenging to manage [35]. Third, *S. acuminata* is amenable to laboratory culture and has been used as a model for studies on dinoflagellate life cycle regulation and stress responses [35,36,37,38,39], providing a foundation for comparative molecular investigations. Despite this, the molecular mechanisms underlying its response to allelochemical stress remain largely unexplored. Therefore, elucidating the response of *S. acuminata* to protocatechuic acid not only addresses this knowledge gap but also provides insights into allelopathic interactions with bloom-forming dinoflagellates, informing the development of targeted HAB control strategies.

Single- and multiomics approaches, based on molecular biology and bioinformatics, have been applied to study algal physiological ecology and biomarker identification [40,41,42,43,44,45,46]. Despite different emphases, these omics approaches aim to understand allelopathic inhibitory mechanisms on algae and algal stress responses. No single technique is suitable for all HAB species, but omics tools (genomics, transcriptomics, proteomics, metabolomics) provide new insights into HAB dynamics [47]. Thus, multiomics research is increasingly applied to algal physiological and biochemical processes, including responses to allelopathy [20,29,48,49], revealing algal response and interaction mechanisms across life stages and environments and offering a broader perspective for understanding these organisms.

In our previous studies, we discovered that protocatechuic acid (PA) could significantly inhibit the growth rate, photosynthetic activity, and affect the microbial community structure around *S. acuminata* [19,50]. Additionally, the half-effective inhibition concentration (EC_50_) of PA was determined in our prior work, yielding reliable results [50]. However, despite these preliminary findings, the full molecular mechanisms underlying PA-mediated inhibition of dinoflagellate growth and the comprehensive molecular response of *S. acuminata* to PA stress remain unclear. Specifically, the key molecular pathways, regulatory networks, and functional biomolecules (genes, proteins, metabolites) involved in the interaction between PA and *S. acuminata* have not been systematically elucidated. To fill this critical research gap, the present study employed *S. acuminata* as the model dinoflagellate and adopted the EC_50_ concentration of PA confirmed in our previous work. Multiple omics techniques were integrated to investigate, at multiple molecular levels, the intrinsic mechanisms of PA’s allelopathic effects on dinoflagellates and the molecular response strategies of *S. acuminata* to PA. This study is expected to further clarify the molecular basis of dinoflagellates’ response to allelochemicals and provide more targeted insights for the ecological control of HABs.

## 2. Results

### 2.1. Transcriptomic Changes in S. acuminata Under Protocatechuic Acid Stress: Widespread Upregulation of Antioxidant and Energy Metabolism Genes

#### Drastic Changes in Primary Energy Metabolism Process in *S. acuminata*

**Figure 1:**
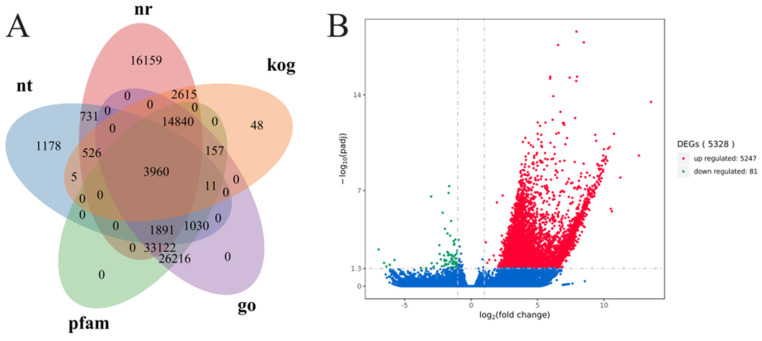
(**A**) Venn diagram illustrating the annotation of unigenes in the nr, nt, kog, pfam, and go databases. (**B**) Volcano plot of DEGs, where red dots represent those upregulated significantly with FC ≥ 2.0, green dots indicate those downregulated significantly with FC ≤ 0.5, and blue dots indicate genes whose expression did not significantly different or the |log2foldchange| < 1. A total of 5328 DEGs were identified across the groups subjected to PA for 48 h and the control for 48 h. The annotation details are shown in Table S3.

The unigenes were compared with those from seven prominent databases, resulting in successful annotation for 63.48% of the unigenes in at least one database (Figure 1A). A total of 5328 significant differentially expressed genes (DEGs) were identified between the groups treated with protocatechuic acid (PA) for 48 h and the control group for the same duration. The creation of a volcano plot indicated that the majority of the DEGs, totaling 5247, were upregulated, whereas only 81 DEGs were downregulated (Figure 1B). Among the 5247 upregulated genes, 741 were not annotated in any database, and of the 81 downregulated genes, 34 also lacked annotation.

**Figure 2:**
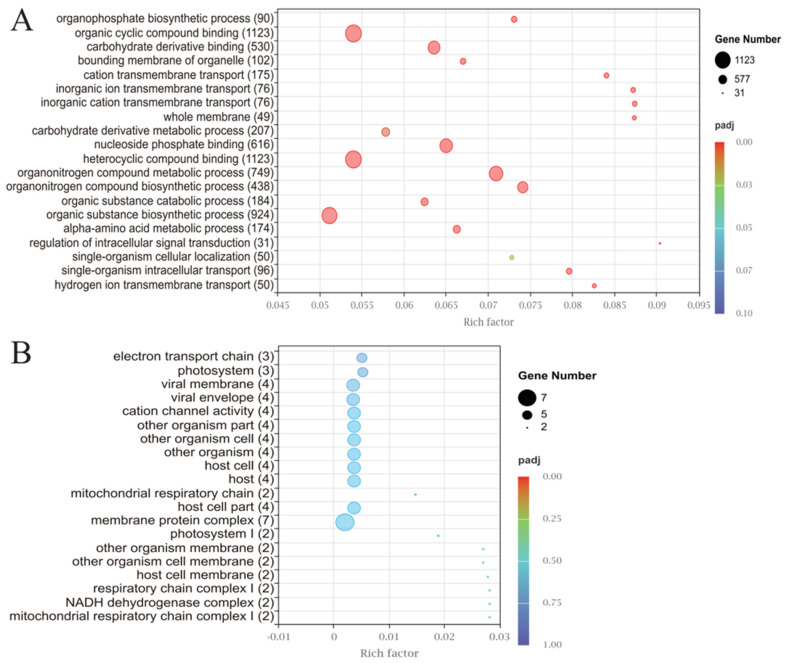
(**A**) The top 20 enriched GO pathways of the upregulated DEGs, with the numbers in parentheses representing the number of upregulated DEGs. (**B**) The top 20 enriched GO pathways of downregulated DEGs, with the numbers in parentheses representing the number of downregulated DEGs.

**Figure 3:**
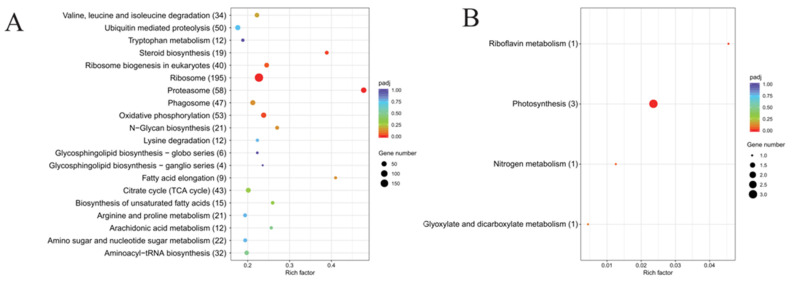
(**A**) The 20 most significantly enriched KEGG tertiary pathways among the secondary pathways of the upregulated DEGs, with the numbers in parentheses representing the number of upregulated DEGs. (**B**) The 20 most significantly enriched KEGG tertiary pathways among the secondary pathways of downregulated DEGs, with the numbers in parentheses representing the number of downregulated DEGs. The size of the bubbles represents the number of DEGs, and the color of the bubbles indicates the significance of enrichment based on padj.

Gene Ontology (GO) analysis of the DEGs revealed three dominant biological themes in response to PA stress (Figure 2A,B). First, upregulated DEGs were predominantly enriched in processes related to energy generation and primary metabolism, including hydrogen ion transmembrane transport, oxidative phosphorylation, and carbohydrate derivative metabolism, indicating an increased cellular demand for energy under stress. Second, a substantial number of upregulated DEGs were associated with transmembrane transport and cellular localization, particularly inorganic cation and ion transport, suggesting active remodeling of ion homeostasis and intracellular trafficking. Third, downregulated DEGs were primarily associated with photosynthesis and related processes, such as light harvesting and photosystem II assembly, pointing to PA-induced impairment of photosynthetic machinery. KEGG pathway analysis corroborated these findings, with significant upregulation of oxidative phosphorylation, ribosome, and proteasome pathways, and downregulation of photosynthesis, riboflavin metabolism, and nitrogen metabolism pathways (Figure 3A,B, Table S4). A total of 2023 DEGs were enriched in 104 KEGG pathways, primarily involving metabolism (931 DEGs), genetic information processing (759 DEGs), and cellular processes (163 DEGs). Collectively, the transcriptomic results demonstrate that PA stress induces coordinated reprogramming of energy metabolism, transport systems, and photosynthetic pathways in *S. acuminata*.

KEGG pathway analysis of DEGs revealed coordinated changes across these pathways that collectively support the allelopathic inhibition hypothesis that PA suppresses algal growth by disrupting core metabolic functions while simultaneously triggering stress defense responses (Figure 3A,B, Table S4). Specifically, the significant upregulation of the oxidative phosphorylation (53 upregulated DEGs), ribosome (195 upregulated), and proteasome (58 upregulated) pathways reflects an enhanced cellular investment in energy production and protein turnover, likely as a compensatory mechanism to counteract PA-induced damage. In contrast, the downregulation of photosynthesis (3 downregulated DEGs), nitrogen metabolism, and riboflavin metabolism pathways provides direct molecular evidence for PA’s inhibitory effect on primary production—a key mechanism underlying growth suppression. Notably, the concurrent upregulation of steroid biosynthesis (19 upregulated) and fatty acid metabolism pathways suggests that *S. acuminata* attempts to remodel membrane lipid composition to maintain cellular integrity under allelochemical stress, consistent with the GO findings on enhanced transmembrane transport. Together, these KEGG results demonstrate that PA exerts its allelopathic effect through dual-mode action: directly suppressing photosynthetic carbon fixation while indirectly imposing energy demands that redirect cellular resources toward stress acclimation, ultimately compromising growth and proliferation.

### 2.2. Proteomic Analysis of S. acuminata Exposed to Protocatechuic Acid: Significant Modulation of Photosynthetic and Lipid Metabolic Pathways

#### 2.2.1. Protein Concentration, Quality and Identification

The protein quantification standard curve had an R^2^ of 0.9866, indicating the high accuracy of the gradient concentration measurements (Figure S3). SDS-PAGE revealed that the extracted proteins were evenly distributed, with no degradation or high-abundance proteins observed and no significant difference in protein amounts between groups, thus demonstrating good repeatability (Figure S4).

**Figure 4:**
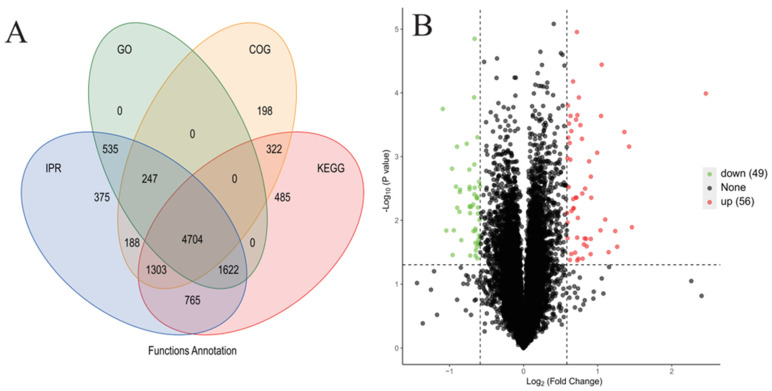
(**A**) Functional annotations of DEPs in the GO, IPR, COG, and KEGG databases. (**B**) Differential expression of proteins, where green dots represent those downregulated significantly with FC ≤ 0.67, red dots represent those upregulated significantly with FC ≥ 1.5, and black dots indicate no significant difference and FC between 0.67 to 1.5.

On the basis of the transcriptomic database for *S. acuminata*, a total of 447,830 secondary spectra were identified, with 130,429 being effective spectra that matched 70,293 peptides, leading to the identification of 13,955 proteins. A total of 68.67% of the total proteins were identified in the four databases (Figure 4A). The remaining 4374 proteins could not be annotated in the database. Whether these unannotated proteins represent lineage- or species-specific proteins remains unclear, as current databases may lack sufficient representation from closely related species. It is also possible that some of these proteins are conserved but diverged beyond recognition. Further investigation including comparative genomics and experimental validation will be necessary to determine the nature and functional significance of these unannotated proteins in *S. acuminata*. The annotations of these proteins were comparable to those of the transcriptomic unigenes.

#### 2.2.2. Functional Annotation of Proteins and Distribution of Differentially Expressed Proteins (DEPs)

A comparison of proteins with a FC ≥ 1.5 or ≤0.67 and a significant difference according to a t test (*p* value ≤ 0.05) revealed that 56 proteins were significantly upregulated at 48 h PA, and 49 proteins were significantly downregulated (Figure 4B). There were 41, 26, and 6 DEPs annotated in the GO, KEGG and IPR databases, respectively. More detailed functional descriptions of these significant DEPs can be found in Table S5.

Functional categorization of the 105 DEPs revealed distinct quantitative patterns between the upregulated and downregulated proteins (Table S5). Among the 56 upregulated proteins, the majority (33 proteins) were associated with metabolic processes, with the largest subcategories being energy metabolism (e.g., malate dehydrogenase, FC = 1.54; fumarate reductase, FC = 1.59) and lipid metabolism (e.g., acyl-CoA dehydrogenase, FC = 1.88 and 1.78; sterol methyltransferase, FC = 1.5). This was followed by proteins involved in genetic information processing and signal transduction (9 proteins), including ribosomal protein S6 (FC = 1.87), proliferating cell nuclear antigen (PCNA, FC = 1.56), and RNA polymerase subunit (FC = 1.51). Notably, 8 upregulated proteins were directly related to redox detoxification and glutathione metabolism (e.g., glutathione S-transferase, FC = 1.68 and 1.52; glutamate–cysteine ligase, FC = 2.06), indicating an active oxidative stress response.

**Figure 5:**
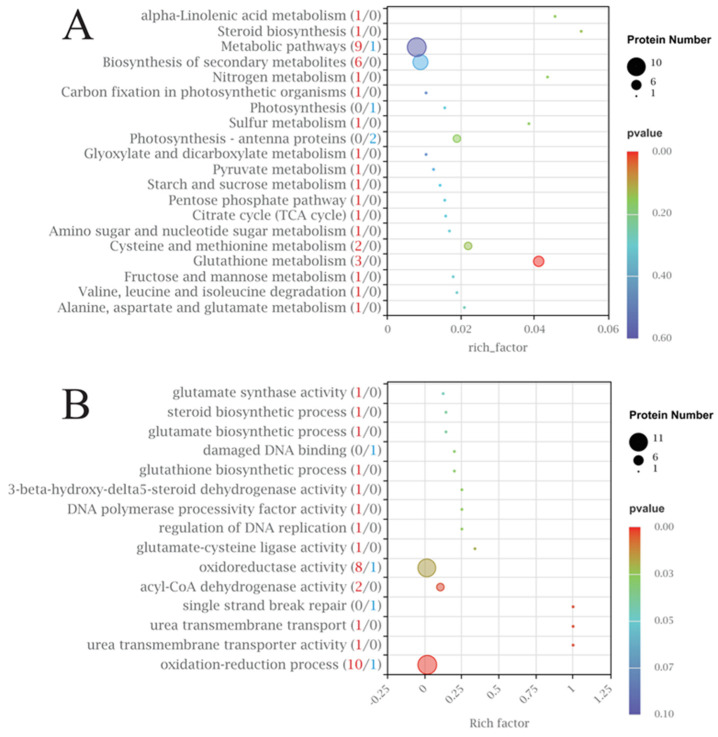
(**A**) KEGG pathway enrichment of DEPs. (**B**) GO pathway enrichment of DEPs. The red numbers in parentheses represent the number of upregulated DEPs, and the blue numbers represent the number of downregulated DEPs.

In contrast, the 49 downregulated proteins showed a markedly different distribution. The downregulated proteins were predominantly involved in photosynthesis, genetic information processing, and signal transduction (Figure 5). The most prominent category was photosynthesis and light harvesting (5 proteins), including light-harvesting complex I chlorophyll a/b binding protein (FC = 0.64 and 0.67), photosystem II oxygen-evolving enhancer protein 3 (FC = 0.52), and fucoxanthin-chlorophyll a/c binding protein F (FC = 0.63). A substantial proportion (11 proteins) were involved in ribosome assembly and translation (e.g., large subunit ribosomal protein L40e, FC = 0.55; 30S ribosomal protein S22, FC = 0.60; ribosomal protein L14E/L6E/L27E, FC = 0.64), suggesting the suppression of protein synthesis machinery. Additionally, signal transduction proteins (6 proteins), including calcium-dependent protein kinase (FC = 0.60), cAMP phosphodiesterase (FC = 0.53), and cAMP-dependent protein kinase regulator (FC = 0.65), were downregulated, pointing to potential disruption of cellular signaling networks. Other downregulated proteins were associated with DNA repair (alkylated DNA repair dioxygenase AlkB, FC = 0.59), chromatin remodeling (lysine-specific demethylase 8, FC = 0.65), and flagellar energy metabolism (creatine kinase, FC = 0.64), representing 8 proteins in total.

This quantitative comparison reveals that PA stress induces asymmetric regulatory responses in *S. acuminata*: while the cell activates metabolic and detoxification pathways to cope with stress (primarily through upregulation), it simultaneously experiences the suppression of photosynthesis, protein synthesis, and signaling processes (primarily through downregulation). This pattern is consistent with a resource reallocation strategy, where energy and resources are diverted from growth-related processes toward stress acclimation and survival. The observed transcriptome–proteome discordance in ribosomal components further suggests that this reallocation may operate at multiple regulatory levels, with transcriptional activation representing an unfulfilled compensatory attempt rather than a functional outcome.

### 2.3. Metabolomic Profiling of S. acuminata Treated with Protocatechuic Acid: Adaptive Remodeling of Lipid and Amino Acid Metabolism for Stress Tolerance

#### 2.3.1. Functional Annotation of Metabolites

A total of 1475 compounds were detected as intracellular metabolites in positive ion mode, whereas 877 compounds were detected in negative ion mode. Among the extracellular metabolites, 197 compounds were detected in positive ion mode, and 106 compounds were detected in negative ion mode. The KEGG, HMBD and LIPID MAPS databases were used to annotate the compounds, and Figures S5–S7 show the annotations of the intracellular and extracellular metabolites in positive and negative ion modes. The identification and fold change of all DEMs are shown in Supplementary File S3.

#### 2.3.2. Analysis and Classification of Differentially Expressed Metabolites (DEMs)

**Figure 6:**
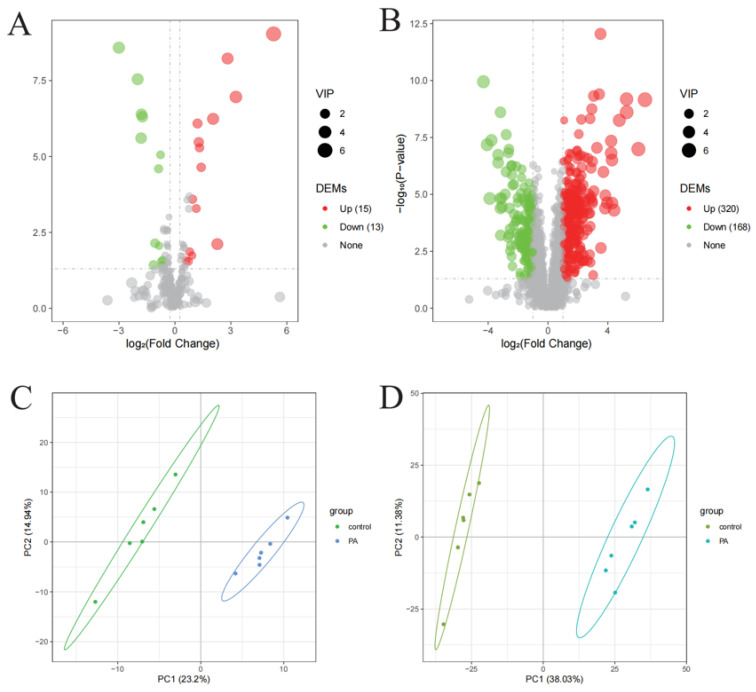
(**A**) The number and expression of DEMs among extracellular metabolites. (**B**) The number and expression of DEMs among the intracellular metabolites. In (**A**,**B**), the size of the bubbles indicates their VIP values, with red dots representing upregulated DEMs and green dots representing downregulated DEMs. The thresholds of DEMs were set for VIP > 1.0, fold change (FC) > 2.0 or FC < 0.5, and *p* value < 0.05. There were 488 and 28 DEMs in the intracellular and extracellular regions, respectively. (**C**,**D**) PLS-DA analysis results of extracellular and intracellular metabolites in the treatment and control groups, respectively.

Figure 6A,B shows how metabolites are regulated and their VIP values in extracellularly and intracellular samples, respectively. The number of upregulated metabolites was greater than that of the downregulated metabolites for both intracellular and extracellular metabolites. Specifically, 320 and 15 DEMs were upregulated intracellularly and extracellularly, respectively.

Figure 6C,D presents the results of PLS-DA, which illustrated the distribution of the two groups of samples in the extracellular and intracellular data. Both figures clearly show a separation between the control and PA groups, indicating that significant differences in metabolomics characteristics were influenced by PA treatment both intracellularly and extracellularly. These results highlight the significant impact of PA treatment on the expression of metabolites, with the PA group showing a distinct metabolic profile compared with the control group.

**Figure 7:**
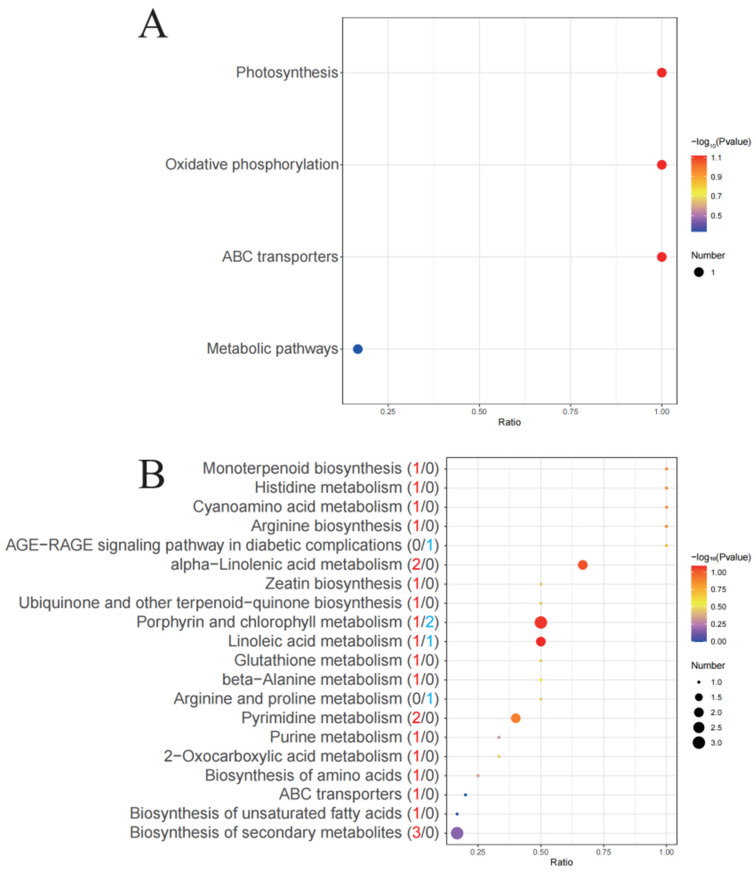
(**A**) KEGG pathway enrichment of DEMs among extracellular metabolites. (**B**) KEGG pathway enrichment of DEMs among intracellular metabolites. The red numbers in parentheses represent the number of upregulated DEMs, and the blue numbers represent the number of downregulated DEMs.

Figure 7 and Table 1 show the results of the KEGG enrichment analysis of the DEMs. KEGG enrichment analysis of intracellular DEMs revealed three dominant metabolic trends in *S. acuminata*’s response to PA stress (Table 1). First, amino acid and nitrogen metabolism emerged as the most prominent category, with multiple pathways affected including arginine, histidine, glutathione, and purine/pyrimidine metabolism. The majority of detected metabolites in these pathways were upregulated (e.g., N-acetylornithine, FC = 2.88; carnosine, FC = 2.28; adenine, FC = 2.06), indicating enhanced nitrogen mobilization and turnover under stress. Second, lipid metabolism underwent significant remodeling, particularly in fatty acid and terpenoid pathways. This included the upregulation of linoleic acid-derived metabolites (13-OxoODE, FC = 2.64) and α-linolenic acid-derived signaling molecules (methyl jasmonate, FC = 3.81; volicitin, FC = 5.18), alongside the downregulation of arachidonic acid precursors (dihomo-γ-linolenic acid, FC = 0.39)—suggesting the reconfiguration of membrane composition and activation of lipid-based stress signaling. Third, photosynthetic pigment metabolism was perturbed, with chlorophyll degradation intermediates (pheophorbide a, FC = 0.30; pyropheophorbide a, FC = 0.16) downregulated while the synthesis intermediate protoporphyrin IX (FC = 4.26) was upregulated, reflecting photosynthetic damage and compensatory attempts to maintain pigment levels. Collectively, these metabolic changes reveal a dual-pronged adaptive strategy: nitrogen mobilization to support stress acclimation and lipid remodeling to maintain membrane integrity and activate stress signaling, with photosynthetic perturbations corroborating the inhibition observed at transcript and protein levels.

The intracellular DEMs were enriched in more metabolic pathways than their extracellular counterparts (Figure 7B), with KEGG annotation revealing a distribution across amino acid metabolism, lipid metabolism, nucleotide metabolism, metabolism of cofactors and vitamins, and metabolism of terpenoids and polyketides (Table 1). These findings demonstrate that PA treatment induces significant reprogramming of multiple intracellular metabolic pathways in *S. acuminata*. Notably, a striking contrast was observed between intra- and extracellular metabolite profiles: while intracellular DEMs were predominantly upregulated (320 out of 488), and extracellular DEMs were largely downregulated (13 out of 28) (Figure 7). This contrasting pattern likely reflects an active retention strategy, whereby stressed cells conserve key intracellular metabolites particularly amino acids, lipids, and antioxidants to support stress acclimation and repair processes while simultaneously reducing the release of these compounds into the surrounding environment to minimize metabolic losses or limit potential autotoxic effects. Alternatively, the downregulation of extracellular metabolites could indicate reduced membrane integrity or impaired secretion systems under PA stress, leading to the decreased export of metabolic products.

**Figure 8:**
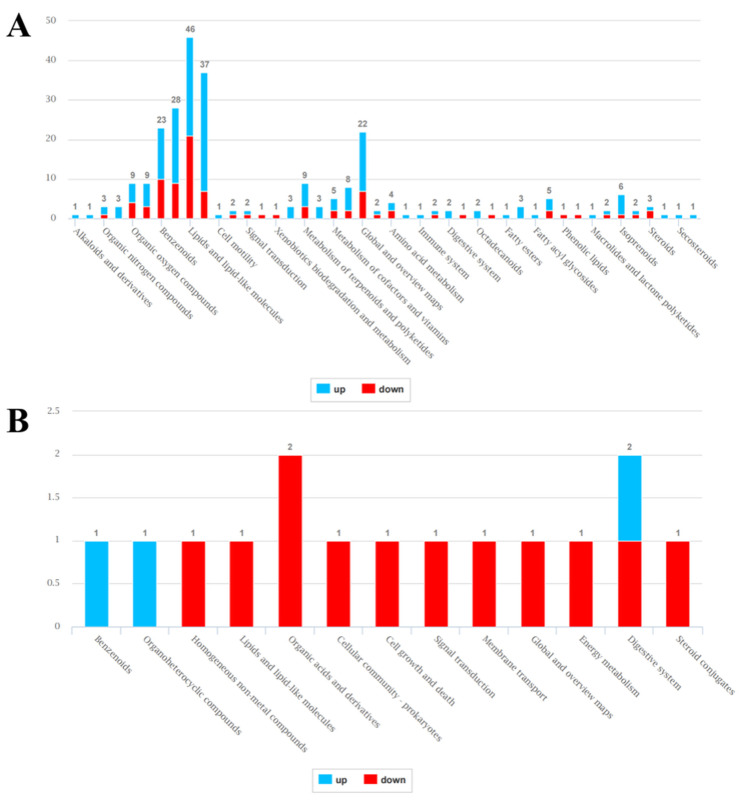
Distribution and number of DEMs in the KEGG, HMBD, and LIPID MAPS databases, where red indicates upregulated DEMs and blue indicates downregulated DEMs. (**A**) Representative intracellular DEMs and (**B**) extracellular DEMs.

Annotation in the KEGG, HMBD, and LIPID MAPS databases revealed that most of the intracellular DEMs belonged to the groups of benzenoids, organoheterocyclic compounds, lipids and lipid-like molecules, organic acids, and derivatives. Most of the DEMs were upregulated, which was consistent with the quantification results (Figure 8A). However, the extracellular DEMs presented different expression patterns after annotation. Most of the extracellular DEMs were downregulated even though their number was much lower than that of the intracellular DEMs (Figure 8B).

## 3. Discussion

**Figure 9:**
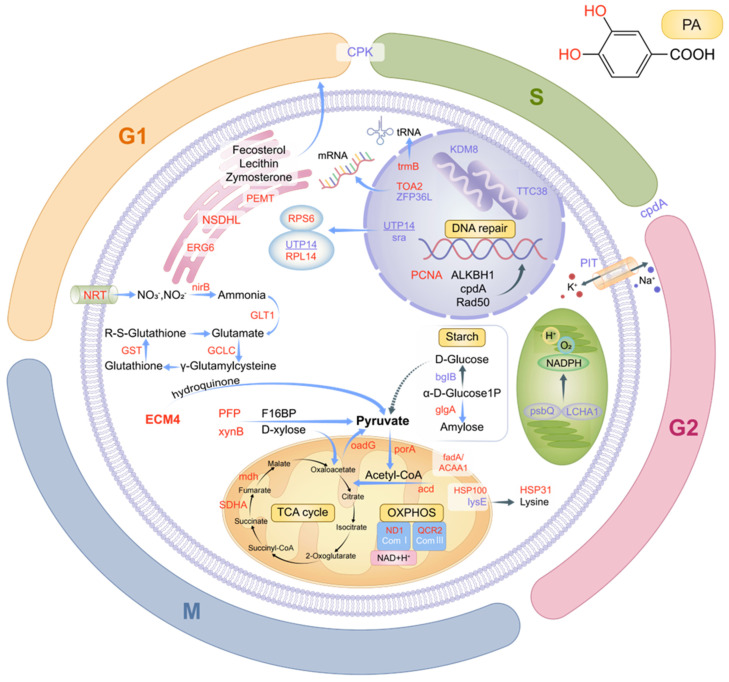
Cellular overview of DEPs and their potential roles in altering the metabolic pathways of *S. acuminata* under PA repression. The red text indicates upregulated proteins, while blue text indicates downregulated ones. The bold text indicates the proteins whose expression patterns are consistent with their corresponding genes in transcriptomic level, while the underlined text indicates the proteins whose expression patterns are opposite to their corresponding genes in transcriptomic level. For a detailed list of abbreviations, refer to Table S7.

The differences in the various omics markers identified in this study indicate that under the stress of protocatechuic acid (an allelopathic substance), the processing of genetic material, energy metabolism, and photosynthesis—which are important life processes of *S. acuminata*—are affected to varying degrees (Figure 9). These changes in important life processes not only help us understand the deeper and multilevel responses of *S. acuminata* to stress, thereby strengthening research on the prevention and control of HABs, but also provide novel insights into the algacidal mechanism and application prospects of allelopathic substances.

### 3.1. Disruption of Genetic Information Processing and Cell Cycle Disturbances

The widespread upregulation of ribosomal protein genes (195 unigenes) and ribosome biogenesis factors (40 unigenes) observed in the transcriptomic data indicate a significant transcriptional response in the protein synthesis machinery of *S. acuminata* under PA stress. However, it is important to note that increased expression of ribosomal genes does not necessarily equate to enhanced or impaired translation. Ribosomal gene transcription is merely one step in the multi-layered process of protein synthesis, which includes mRNA processing, export, ribosome assembly, translation initiation, elongation, and termination—each subject to independent regulation. The observed transcriptional upregulation could represent: (i) a compensatory response to impaired ribosome function or reduced translational capacity; (ii) a stress-induced metabolic shift requiring increased production of specific ribosomal components; or (iii) non-specific transcriptional activation unrelated to actual translational output. Consistent with this cautious interpretation, previous studies on dinoflagellates under environmental stress have reported both up- and downregulation of ribosomal genes, with expression patterns varying depending on the specific stressor and species [51,52,53,54], suggesting that ribosomal gene expression alone is not a reliable proxy for translational activity. Therefore, while our transcriptomic data point to transcriptional reprogramming of the translation machinery, drawing conclusions about actual translational efficiency or disruption would require complementary analyses—such as polysome profiling or ribosome footprinting—that directly measure ribosome engagement and protein synthesis rates.

However, unlike the hundreds of upregulated unigenes in the ribosome related pathway, only several DEPs associated with genetic material processing presented significant differences and different regulatory patterns, indicating a potential discrepancy between the expression of proteins related to genetic information processing. For example, almost all DEGs related to ribosomal subunit proteins and proteins related to ribosome biogenesis were significantly upregulated, whereas only five ribosomal proteins were differentially expressed (Table 1), not only in the difference of quantity but also in expression patterns. In our results, most DEPs related to ribosomal proteins were downregulated, including large subunit ribosomal protein L40e, ribosomal protein L14E/L6E/L27E/60S ribosomal protein L14, ribosomal L29e protein family, and 30S ribosomal protein subunit S22 family, with fold-change values ranging from 0.55 to 0.64. Only ribosomal protein S6 showed upregulation (FC = 1.87). The ribosomal proteins have functions not only in ribosome composition but are also involved in various regulation processes through translation process. The downregulation of ribosomal proteins could disrupt the translation and cellular regulation. In detail, the downregulation of the large subunit ribosomal protein L40e and the 30S ribosomal protein subunit S22 family could have led to a negative impact on the function of the ribosome in the present study, which led to a decrease in protein synthesis efficiency, cell growth and developmental regulation [55,56]. Therefore, to alleviate these effects and respond to stress from PA, *S. acuminata* may also increase the expression of the ribosomal protein S6. The upregulation of enzymes related to synthesis and repair in the results of this study may indicate that the genetic material of *S. acuminata* was damaged under PA. For instance, studies have noted that the proliferating cell nuclear antigen (PCNA) is associated with cell repair and growth in *Dunaliella salina* (Chlorophyta) [57] and base excision repair functions in dinoflagellates [58].

Regarding RNA, the U3 small nucleolar RNA-associated protein, which is universally present in eukaryotes, is involved in the processing of pre-18S ribosomal RNA in the nucleus of all biological cells. The research showed that the pre-18S ribosomal RNA is part of the complex that activates pre-rRNA processing, and its downregulation hampers the production of 18S rRNA [59]. Moreover, both the downregulated expression of tetratricopeptide repeat protein 38 (TRP), which was noted to possibly be related to chloroplast stability and/or translational machinery and are part of several protein complexes, including RNA in *Chlamydomonas reinhardtii* (Chlorophyta) [60]. The lysine-specific demethylases shape multiple aspects of plant biology, such as flowering time, root architecture, circadian rhythms, DNA-damage responses, pathogen defense, and regeneration while also reinforcing the methylation of transposons and other repetitive elements that lie near protein-coding genes in *Arabidopsis thaliana* (Magnoliophyta) [61]. Therefore, the downregulation of TRP proteins and lysine-specific demethylases could result in incomplete RNA structures and disordered gene coding in *S. acuminata*.

Zinc finger proteins in *S. acuminata* are also affected by PAs. This family of proteins plays crucial roles in gene regulation, influencing many aspects of plant growth and development, and is vital for many cell functions, including transcription regulation, translation, DNA or RNA binding, protein folding, cytoskeleton organization, cell adhesion, and signaling pathway control through protein–protein interactions [62]. PA can also inhibit certain DNA repair enzymes/proteins in *S. acuminata*, such as alkylated DNA repair oxygenases (AlkB). AlkB was found to repair DNA damage caused by alkylating adducts [63]. Additionally, the DNA repair protein Rad50 is also inhibited, and efficient DNA repair in eukaryotes is fundamental for maintaining the integrity and continuity of genetic information in dinoflagellate [64]. The reduction in DNA repair enzymes/proteins in *S. acuminata* under the influence of protocatechuic acid may trigger a series of negative effects.

In addition to the DEPs above-mentioned, some DEPs involved in signal transduction presented upregulation. For example, the upregulation of Ras-related protein activators could stimulate their GTPase activity. The Ras signaling pathway plays crucial roles in controlling various processes, such as cell migration, shape, proliferation and differentiation, apoptosis, survival, and movement, and is associated with responses to a wide range of environmental stresses in plants [65]. The harmful dinoflagellate *Prorocentrum cordatum* (formerly *Prorocentrum minimum*) typically activates the Ras signaling pathway in response to copper sulfate stress [52,66]. The activation of certain proteins in the Ras pathway under PA stress suggests that the Ras signaling pathway may play a role in the response process of *S. acuminata*. In detailed, Ras signaling is directly linked to cell proliferation control. In our study, the concurrent downregulation of cell cycle-related proteins—cAMP phosphodiesterase (FC = 0.53) and calcium-dependent protein kinase (FC = 0.60)—suggests the disruption of normal cell cycle progression. Thus, Ras activation may represent a stress-induced attempt to maintain cell cycle progression, an effort that ultimately fails under sustained PA stress, contributing to the observed growth inhibition. Second, Ras signaling is implicated in the decision between cell survival and programmed cell death. It is plausible that Ras activation may contribute to a survival-oriented cellular decision, steering cells away from cell death and toward cyst formation as a dormancy strategy under persistent stress. In summary, Ras signaling may serve as a hub linking allelochemical stress perception to downstream phenotypic outcomes—growth inhibition and cyst formation—in *S. acuminata*.

Additionally, the present study observed the formation of resting cysts in *S. acuminata* under PA stress (Figure S8), coinciding with the differential expression of several proteins linked to cell cycle regulation and programmed cell death in other organisms. For example, Vardi A et al. found that the downregulation of cysteine proteases can prevent programmed cell death, and in dinoflagellates, the downregulation of cysteine proteases can lead to cyst formation in the dinoflagellate *Peridinium gatunense* [67]. The cyclic AMP phosphodiesterase can catalyze the degradation of cAMP to 5′-AMP, thereby regulating the cAMP signaling pathway in cells. This pathway is crucial in higher eukaryotes for controlling the G1/S and G2/M transitions of the cell cycle [68]. The activity level of cyclic AMP phosphodiesterase is often positively correlated with algal cell abundance in *Ostreopsis* cf. *ovata* [69]. Studies have shown that cAMP signaling in the cell cycle of *Amphidinium operculatum* is consistent with the control of the cell cycle by cAMP in higher eukaryotes [70], and noted that when the activity and/or concentration of cyclic AMP phosphodiesterase is reduced, the cell cycle process between the G1/S and G2/M transitions is inhibited, thereby inhibiting cell division and preventing normal density growth. Like cyclic AMP phosphodiesterase, calcium-dependent protein kinases are also associated with cell cycle regulation and progression [71]. Additionally, research indicates that the downregulation of calcium-dependent protein kinases can lead to a decrease in the growth rate and a stall in the G1 phase of the cell cycle in *Alexandrium catenella* under phosphate limitation [72]. While the observed downregulation of these proteins in *S. acuminata* is consistent with suppressed cell proliferation and the onset of cyst formation, the current data cannot establish a direct causal link. Further functional studies are needed to determine whether these molecular changes actively induce encystment or are part of a broader stress response.

### 3.2. The Alteration of Lipid and Carbohydrate Metabolism

Lipids and sterols are critical components of eukaryotic cell membranes, serving as energy sources and signal transduction mediators. Under environmental stress, changes in membrane sterol composition can enhance stress tolerance [73]. Transcriptomic data revealed widespread upregulation of lipid metabolism pathways, including steroid biosynthesis (19 unigenes), fatty acid elongation (9 unigenes), and unsaturated fatty acid biosynthesis (15 unigenes) (Table S4).

Proteomic analysis confirmed these observations, showing the upregulation of enzymes involved in both lipid synthesis and degradation. Sterol methyltransferase (upregulated), which catalyzes the production of membrane-stabilizing sterols (fucosterol, ergosterol), is commonly associated with enhanced stress tolerance [74,75]. Phospholipid methyltransferase, also upregulated, participates in phosphatidylcholine synthesis—the most abundant membrane phospholipid with additional roles in signal transduction and metabolic regulation [76]. Cytochrome b5 upregulation may influence unsaturated fatty acid content, potentially modulating membrane fluidity [77].

Concurrently, upregulation of acyl-CoA dehydrogenase and acetyl-CoA acetyltransferase suggests enhanced fatty acid β-oxidation, likely providing alternative energy substrates under photosynthetic impairment [78,79,80]. This simultaneous upregulation of both synthetic and degradative pathways may appear contradictory but likely reflects compartmentalized metabolic adjustments: β-oxidation in peroxisomes/mitochondria generates energy, while lipid synthesis in plastids/ER remodels membrane composition for improved stress tolerance.

Metabolomic profiling supported these findings, revealing the accumulation of saturated lipid species alongside the depletion of unsaturated metabolites and phosphatidylcholine degradation products (glycerophosphocholine, lysoglycerophosphocholine). The reduction in phospholipid degradation products, despite unchanged total phospholipid levels, suggests enhanced membrane stability—possibly mediated by altered sterol and fatty acid composition that limits phospholipase accessibility [81,82]. The shift toward saturated lipid metabolites is consistent with membrane reinforcement against oxidative damage, as saturated fatty acids exhibit reduced susceptibility to lipid peroxidation.

Notably, upregulation of methyl jasmonate (FC = 1.9) may activate stress signaling pathways regulating secondary metabolite synthesis [83,84,85], while increased phytol-7 could enhance membrane antioxidant capacity [86]. Together, these coordinated adjustments—energy generation via lipid catabolism and membrane stabilization via lipid remodeling—represent an integrated adaptive strategy to PA stress.

In addition to enhancing the energy supply through increased lipid metabolism, *S. acuminata* also upregulates enzymes related to carbohydrate metabolism in response to protocatechuic acid. Among these enzymes, in xylan 1,4-beta-xylosidase (FC = 1.73), the breakdown of xylan into xylose can be converted to pyruvate and alpha-ketoglutarate to enter the tricarboxylic acid (TCA) cycle for energy production. The upregulation of granule-bound starch synthase could lead to an increase in amylose, resulting in increased starch synthesis. These results indicate that under the stress of protocatechuic acid, *S. acuminata* has potential to accumulate starch. The same mechanism was also observed in Prorocentrum lima under nitrogen limitation [87]. Moreover, the upregulation of 6-phosphofructokinase, a key enzyme in glycolysis, indicates an increase in glycolytic activity for energy demand. An enhancement in the glycolysis pathway also appeared in *Microcystis aeruginosa* exposed to tannic acid and *Thalassiosira pseudonana* (Mediophyceae) under allelopathy from *Karenia brevis* (Dinoflagellata) for cellular energy supply [42,80].

The downregulation of β-glucosidase in carbohydrate metabolism can lead to a reduction in glucose availability, thereby affecting glycolysis, the TCA cycle, and the pentose phosphate pathway due to insufficient substrates or intermediates. This can result in a lack of essential cellular energy and NADPH. The downregulation of phosphopantetheine adenylyltransferase and creatine kinase can directly lead to a reduction in coenzyme A (CoA). However, dinoflagellates contain various fatty acids and polyketides. A decrease in CoA could lead to insufficient fatty acid synthesis and reduced ATP production in flagella [88,89]. The scarcity of intermediates, energy, and NADPH may also lead to metabolic compensation in energy production pathways, as indicated by the upregulation of numerous proteins related to metabolism, as above-mentioned.

### 3.3. Amino Acid and Nitrogen Metabolism

At the proteomic level, upregulated nitrogen uptake-related genes such as NAD(P)H-nitrite reductase, large subunit (NR, FC = 1.55) and nitrate transporter (NRT, FC = 1.74) could enhance nitrogen uptake and remobilization, which is consistent with the results in the metabolite features. At the metabolite level, the enrichment and synthesis of amino acids are common metabolic responses of organisms to stress [90]. In this study, the upregulated amino acids related metabolites included carnosine, DL-2,6-Diaminopimelic acid, N-Acetylornithine, Ala-Met, Leucylproline, L-Asparaginyl-L-threonine, Ser-Gly, Asp-lys, Gly-Leu, Asp-Arg, L-Theanine, and Ala-Leu (Table S5). Similar results were found in *Neopyropia yezoensis* (formerly *Pyropia yezoensis*) (Rhodophyta), which suffered from okadaic acid [91]. The upregulation of these genes and proteins may also indicate increased nitrogen demand in *S. acuminata*.

The upregulation of glutamate synthase (FC = 1.70) indicates an increase in glutamate synthesis. Glutamate can be converted to succinate to participate in the TCA cycle, thereby affecting the pathway and reflecting the increased energy requirement of *S. acuminata*. Glutathione S-transferases (GSTs, FC = 1.68 and 1.52), which are widely distributed in eukaryotes and prokaryotes, are not only involved in amino acid metabolism but also act as isoenzymes for the detoxification of endogenous and exogenous toxic substances. The overlap in function between GSTs and peroxidases (peroxiredoxins and glutathione peroxidases) suggests their important role in alleviating oxidative stress in organisms [92], whereas glutathione plays a significant role in protein modification and cell signaling [93]. The upregulation of gamma-glutamylcysteine synthetase, which links cysteine and glutamate to the production of glutathione, is likely to increase the glutathione levels. The upregulation of enzymes in this system under the influence of PA suggests that *S. acuminata* may be responding to pro-oxidant conditions induced by PA, though direct measurements of ROS would be needed to confirm oxidative stress.

Glutathionyl-hydroquinone reductase (FC = 1.88), which is widely found in bacteria, fungi, and plants, can promote the deglutathionylation of quinones through catalysis by cysteine [94]. Glutathionyl-hydroquinone is more prone to autoxidation than quinones, leading to an increase in ROS, which adversely affects cells [95]. The enzyme converts glutathionyl-hydroquinone back to quinones, allowing them to re-enter metabolic pathways, reducing their cytotoxic effects, and increasing metabolic efficiency. Nucleoside diphosphate kinase (FC = 1.732), which transfers the terminal phosphate group from (d)NTPs (primarily ATP) to any (d)NDP to produce (d)NTPs, also participates in signal transduction [96]. The upregulation of nucleoside diphosphate kinase helps plant cells overcome the oxidative stress caused by methylene blue, cold, heat, and high salinity stresses [97,98]. Thus, the upregulation of glutathionyl-hydroquinone reductase and nucleoside diphosphate kinase may contribute to mitigating potential pro-oxidant effects of PA, consistent with an adaptive response to oxidative challenge, and the widespread upregulation of amino acids indicates their role in the regulation of metabolic processes and signaling pathways in response to abiotic stress in surviving *S. acuminata* cells.

Possible reasons for this phenomenon include the following. (1) Amino acids can serve as important energy sources when sugar energy is insufficient. The body requires more energy to defend against and survive abiotic stress, leading to the large-scale synthesis of amino acids as energy storage substances [99,100,101]. The upregulation of nitrogen assimilation-related proteins (e.g., nitrate transporters, nitrate reductase) at the transcriptomic and proteomic levels, together with the increased expression of enzymes in central energy metabolism pathways (e.g., glycolysis, TCA cycle), supports this explanation. (2) Protocatechuic acid may disrupt the osmotic balance of *S. acuminata* cells. Amino acids act as organic osmolytes and play a critical role in osmoregulation during osmotic imbalance [102]. (3) Beyond these canonical roles, certain amino acids may participate in regulatory signaling. Notably, recent evidence indicates that algae can produce the hormone auxin (indole-3-acetic acid) from tryptophan [103], and tryptophan itself can trigger responses overlapping with auxin signaling [104]. Thus, the widespread amino acid accumulation observed here might also reflect hormone-mediated stress acclimation, though this possibility requires direct experimental validation in *S. acuminata.*

The regulation of other nitrogenous compounds, such as amines, amides, and alkaloids, under protocatechuic acid stress suggests nitrogen redistribution in *S. acuminata*, thus emphasizing the importance of these nitrogenous metabolites for cellular homeostasis, which is consistent with transcriptomic data (Table S4). Despite studies showing that diatoms under oxidative stress may experience reduced nitrogen absorption capacity due to changes in the redox state of key nitrogen absorption enzymes or a reduction in reductive substances (such as NADPH) [105], the nitrogen absorption capacity of *S. acuminata* was enhanced under the influence of protocatechuic acid. This enhancement might occur because many nitrogen-containing compounds (such as amines and amino acids) can also act as scavengers of free radicals and osmolytes [106]. These findings are consistent with a cellular response to both osmotic and pro-oxidant challenges caused by PA, though confirmatory physiological measurements are warranted.

### 3.4. Photosynthesis

The DEGs in photosynthesis showed downregulation (psbB, psaA, psaB). The downregulation of the photosynthesis pathway in *S. acuminata* was consistent with some other studies. Tao et al. found that malonic acid could inhibit the transcription of genes (*rbcL*, *psbA1*) in *Microcystis aeruginosa* (Cyanobacteriophyta) [107]. The gene expression of photosynthesis in dinoflagellate *Prorocentrum obtusidens* (formerly *Prorocentrum donghaiense*) was inhibited by extracellular chemicals released by *Karlodinium veneficum* (Dinoflagellata) [108]. Present study on the regulation of photosynthesis at the transcriptomic level indicates that PA causes the downregulation of several genes in PSI and PSII of *S. acuminata*, which might result in damage to the integrity of the chloroplast structure.

The inhibition by protocatechuic acid also affects proteins related to the photosynthesis pathway. The downregulation of light-harvesting complex I chlorophyll a/b binding protein 1 (FC = 0.64 and 0.67), which serves as an antenna protein for PSI to transfer energy, may impair chloroplast membrane function and decrease photosynthesis, as observed in the current study. PA also inhibits the expression of photosystem II oxygen-evolving enhancer protein 3 (FC = 0.52) as well as fucoxanthin-chlorophyll a/c binding protein F (FC = 0.63). The downregulation of these proteins may lead to the impaired structure and function of PSII. The reduced content of these proteins in the treatment group suggests that PA has a direct effect on chloroplasts. The inhibition of photosynthesis in microalgae was also found in other studies. For instance, Kuzminov et al. found that a similar situation occurs with algae under heavy metal stress, where light-harvesting complexes were reduced at both the transcriptional and protein levels in *Symbiodinium* spp. (Dinoflagellata) [109]. Li et al. demonstrated that allelochemical p-coumaric acid could inhibit several proteins such as psbA (Photosystem II protein D1), psbC (Chlorophyll a/b binding light-harvesting protein), petF (Ferredoxin), psbD (Photosystem II D2 protein), and psbL (Photosystem II reaction center protein L) in the photosynthesis of *Limnothrix* sp. (Cyanobacteriophyta) through the proteomic approach [20]. Interestingly, ferredoxin was shown to be upregulated (FC = 2.68 and 1.79) under PA stress while the other DEPs related to photosynthesis showed downregulation. A possible explanation of this phenomenon is that ferredoxin protein is critical to photosynthesis related to photosynthetic electron transport and could help to keep the necessary photosynthetic electron flow under antibiotic stress [110]. Even though there were diverse expressions in proteins of different microalgae, they all showed depression under allelochemical stress.

The metabolomics data indicated an increase in the concentration of oxidized phylloquinone (phylloquinone oxide, FC = 2.47) under the influence of protocatechuic acid. The phylloquinone in photosystem I (PSI) was an important electron carrier and electron acceptor for the formation of protein disulfide bonds [111]. The increase in oxidized phylloquinone might be due to its reduced conversion back to phylloquinone, leading to insufficient electron transfer and consequently reducing the activity of the PSII reaction center, which impacts the photosynthetic activity of *S. acuminata*. Moreover, a decrease in hydroquinone and its derivatives, including compounds extracted from the brown alga *Dictyopteris undulata* that are considered to have antioxidant properties [112], was observed following treatment with PA. Therefore, the decrease in hydroquinone content after protocatechuic acid treatment could potentially contribute to increased ROS susceptibility, though this remains to be directly validated.

To prevent further oxidative damage caused by free radicals, plant cells can increase their antioxidant levels by increasing the levels of porphyrin, an intermediate in chlorophyll biosynthesis [113,114]. Compared with the results of the control group, this study revealed an increase in protoporphyrin IX and a decrease in uroporphyrinogen III, with uroporphyrinogen III being a precursor to protoporphyrin IX. Thus, its decrease is likely due to enhanced conversion to protoporphyrin IX. Together with the elevation of protoporphyrin IX under ROS accumulation, the findings of this study indicate that *S. acuminata* can mitigate oxidative damage by increasing protoporphyrin IX levels.

Pheophorbide a (FC = 0.29), pyropheophorbide a (FC = 0.16), and 13(2)-carboxylpyropheophorbide a (FC = 0.11) were significantly downregulated under PA stress (Supplementary materials S3). The results were partially similar with *Karenia mikimotoi* (Dinoflagellata) under high linoleic acid [29], which further proved that PA affected the synthesis of metabolites related to photosynthetic pigments. Pheophorbide a is an important product of chlorophyll a degradation in the marine environment, and its derivatives are typically formed in aging algal cells. The downregulation of chlorophyll degradation products may be due to changes in lipid metabolite composition, leading to increased thylakoid membrane tolerance.

## 4. Materials and Methods

### 4.1. Algal Culture and Experimental Design

*Scrippsiella acuminata* (Ehrenb) Kretschmann et al. (Dinophyceae) (previously identified as *Scrippsiella trochoidea* (Stein) Loeblich III 1976) was purchased from Shanghai Guangyu Biological Technology Co., Ltd. (Shanghai, China) and cultured in F/2 media supplemented with artificial sea salt to achieve a salinity of 29 ± 1 psu. The temperature was set to 20 ± 1 °C, and the light intensity was set to 100 μmol m^−2^s^−1^ with a light–dark cycle of 12 h:12 h. Gentle shaking was performed three times a day. *S. acuminata* in the exponential growth phase were exposed to protocatechuic acid (0.20 mM) for 48 h (treatment group labeled PA) and cultured under normal conditions for 48 h (control group). The 48 h exposure duration was chosen based on our previous studies demonstrating maximal PA inhibitory effects at this time point [19,50], ensuring consistency across our research program and allowing for direct comparison with previously published physiological data. Both groups were analyzed in duplicate at least three times.

### 4.2. Transcriptomic Analysis

The algal cells were collected, frozen in liquid nitrogen, and stored at −80 °C. RNA was extracted using a TRIzol reagent kit (Promega, Madison, WI, USA), and its purity and integrity were assessed. The raw sequencing data were filtered and assembled using Trinity (v2.4.0) and Corset (v4.6). The unigene database was constructed from the longest sequence of each cluster. Gene functional annotation was conducted against seven databases. RSEM (v1.2.15) and DEGSeq (v1.12.0) were used for mapping and differential analysis, respectively. Significant differential expression was determined by padj < 0.05 and |log2foldchange| > 1. Enrichment analysis was performed using GOseq (v1.10.0) and KOBAS (v2.0.12).

The transcriptomic samples were subjected to several quality assessments. First, the results of the gel electrophoresis revealed that the bands were clear without obvious degradation (Figure S1). The RIN for all samples was >5, and the OD260/280 ratio was >1.8 (Table S1). The average number of clean reads per sample reached 12.95 Gb; the Q20 and Q30 values were greater than 98.0% and 95.0%, respectively; and the average GC% of the samples was 62.04%, indicating that the sample quality met the requirements for further assembly and analysis. The data are summarized in Table S2.

A total of 292,330 transcripts and 161,902 unigenes were obtained through Trinity assembly. The length distribution is shown in Figure S2. The transcript and gene N50 values were 1641 and 1646, respectively. An N50 greater than 1000 at transcript concatenation indicated that the assembly strategy was feasible.

### 4.3. Proteomic Analysis

The algal cells were collected, frozen, and lysed using a protein lysis mixture. The supernatant was collected, treated with dithiothreitol and iodoacetamide, and precipitated with acetone. The protein concentration was measured via a Bradford protein quantification kit. Proteins were digested with trypsin and labeled with TMT reagent. The peptides were separated using a C18 column and analyzed by a Q Exactive HF-X mass spectrometer (Thermo Fisher, Waltham, MA, USA). Shotgun proteomics analyses were performed in data-dependent acquisition mode. The raw data were imported into Proteome Discoverer 2.2 for database searching and quantification. DEPs were identified by t tests (*p* ≤ 0.05 and FC ≥ 1.5 or FC ≤ 0.67). GO and KEGG pathway enrichment analyses were conducted to identify significant functions and pathways.

### 4.4. Metabolomic Analysis

The algal cells and extracellular culture medium samples were collected, frozen, and stored at −80 °C. Metabolites were extracted via a methanol solution and filtered for LC-MS analysis. Chromatographic analysis was conducted using a Hypersil Gold column (C18) (Thermo, USA). Mass spectrometry scanning was performed in both positive and negative ion modes. The compounds were identified via molecular and fragment ions and compared against the mzCloud and Chemspider databases. Partial least squares discrimination analysis (PLS-DA) was used to construct a model of metabolite expression levels and sample categories. VIP values, fold change (FC), and t test *p* values were used to screen for differentially abundant metabolites (VIP > 1.0, FC > 2.0 or FC < 0.5, and *p* < 0.05). Enrichment analysis was performed using KEGG pathway analysis to identify significant metabolic pathways.

The detailed methods are provided in Supplementary Materials S4.

## 5. Conclusions

This study elucidated the complex mechanisms underlying the response of the dinoflagellate *S. acuminata* to allelochemical protocatechuic acid, revealing significant alterations at the transcriptomic, proteomic, and metabolomic levels. These findings underscore the multifaceted impact of allelochemical stress on key cellular processes, including genetic material processing, energy metabolism, and photosynthesis. Transcriptomic analysis indicated that protocatechuic acid induces the upregulation of genes involved in antioxidant systems and enzymes related to mitochondria, electron transport, and oxidative phosphorylation, suggesting that *S. acuminata* attempts to counteract the oxidative stress induced by allelochemicals. However, the lack of correlation between the transcriptome and proteome implies a reliance on post-translational regulation in dinoflagellates, highlighting the complexity of stress response regulation. Proteomic results revealed that protocatechuic acid affects proteins associated with photosynthesis, energy metabolism, and genetic information processing. The upregulation of certain proteins indicates increased energy supply through lipid metabolism, whereas the downregulation of other proteins—particularly those involved in photosynthesis—suggests potential inhibition of the photosynthetic system. Metabolomic profiling further supported these findings, revealing shifts in lipid and amino acid metabolism indicative of an adaptive strategy for energy storage and the maintenance of cellular homeostasis under allelochemical stress. Notably, while the multiomics approach employed herein provides a comprehensive molecular framework for understanding *S. acuminata*’s response to protocatechuic acid, the current conclusions were primarily derived from omics-level data. Integrating foundational biological metrics in future work would strengthen the mechanistic conclusions and provide more comprehensive validation, including supplementary biochemical assays, physiological measurements, and ultrastructural observations to bridge molecular alterations with functional and structural phenotypes. Overall, this study provides insights into the molecular mechanisms underlying the allelopathic effects of protocatechuic acid on *S. acuminata*, contributing to a deeper understanding of algal responses to allelochemical stress and offering implications for the development of novel, environmentally friendly strategies for the control of HABs. Furthermore, this work supports the utility of multiomics approaches in HAB research while emphasizing the importance of integrating foundational biological metrics to enhance the rigor and translational potential of future studies.

## Figures and Tables

**Table 1:** Metabolic pathways and distribution of intracellular DEMs in *Scrippsiella acuminata* (PA 48 h vs. control 48 h).

Category	Metabolic Pathway	Name of DEMs	Fold Change	Up/Down	Function Description
Environmental Information Processing	ABC transporters	Romicil	0.499	Down	A macrolide antibiotic. Upstream product: Malonyl-CoA.
Amino acid metabolism	Arginine and proline metabolism	Norspermidine	0.073	Down	One of the final products in arginine and proline metabolism. Upstream product: Carboxynorspermidine.
	Arginine biosynthesis, 2-oxocarboxylic acid metabolism	N-Acetylornithine	2.882	Up	A key intermediate in the biosynthesis of arginine. Upstream product: Glutamate. Downstream product: Citrulline and ornithine.
	Cyanoamino acid metabolism	Dhurrin	3.453	Up	One of a cyanogenic glucosides derived from thyrosine serves as a nitrogen storage compound, providing protection against biotic stress and helps plants resist environmental stresses. Upstream product: L-Tyrosine. Downstream product: Hydrogen cyanide.
	Glutathione metabolism	Homotrypanothione	2.224	Up	A glutathione analogue involved in the cellular antioxidant defense system. Upstream product: Glutathione. Downstream product: Homotrypanothione disulfide.
	Histidine metabolism, beta-Alanine metabolism	Carnosine	2.281	Up	A key intermediate product of histidine metabolism and beta-alanine metabolism. Upstream product: L-histidine, beta-alanine. Downstream product: Histidine and anserine.
Lipid metabolism	Biosynthesis of ubiquinone and other terpenoid-quinones	Vitamin K1 epoxide	2.469	Up	A key intermediate in the vitamin K cycle, interconversion with phylloquinone. Upstream product: Phylloquinone. Downstream product: Phylloquinone.
	Linoleic acid metabolism	Dihomo-gamma-linolenic acid	0.385	Down	An important precursor of arachidonic acid, which is the raw material of arachidonic acid metabolism. Upstream product: Linolenic acid. Downstream product: Arachidonic acid.
		13-OxoODE	2.641	Up	One of the final product in linoleic acid metabolism. Upstream product: 13(S)-HPODE.
	α-Linolenic acid metabolism	Volicitin	5.179	Up	One of the final products in α-linolenic acid metabolism. Upstream product: α-Linolenic acid.
		Methyl jasmonate	3.809	Up	One of the final products in α-linolenic acid metabolism. Upstream product: Jasmonic acid.
Nucleotide metabolism	Purine metabolism, Zeatin biosynthesis	Adenine	2.061	Up	A key intermediate in purine metabolism and a component of ATP. Upstream product: ADP, AMP. Downstream product: Hypoxanthine, ATP.
	Pyrimidine metabolism	dUDP	2.160	Up	One of the precursors of DNA synthesis. Upstream product: UDP. Downstream product: dUMP.
		Deoxyuridine	2.200	Up	Participates in DNA synthesis and is a precursor for DNA and helps maintain genomic stability and cell proliferation. Upstream product: dUDP. Downstream product: dUMP, uracil, deoxyribose-1-phosphate.
Metabolism of terpenoids and polyketides	Carotenoid biosynthesis	Astaxanthin	0.392	Down	An important product in Astaxanthin biosynthesis, plays a role as antioxidant. Upstream product: Canthaxanthin and adonixanthin. Downstream product: Astaxanthin diester.
	Monoterpenoid biosynthesis	Loganin	13.006	Up	A secondary metabolite and important precursor of secologanin, which further participates in indole alkaloid biosynthesis for enhancing the stress resistance. Upstream product: Geraniol. Downstream product: Secologanin.
Energy metabolism/Transport	Oxidative phosphorylation, Photosynthesis, ABC transporters	Phosphate	0.288	Down	A central metabolite involved in numerous cellular processes including energy metabolism (ATP synthesis), photosynthetic phosphorylation, and phosphate transport. Its downregulation may reflect altered energy status or transport dynamics, though its broad roles preclude pathway-specific mechanistic conclusions.
Metabolism of cofactors and vitamins	Porphyrin and chlorophyll metabolism	Protoporphyrin IX	4.260	Up	An intermediate in chlorophyll synthesis. Upstream product: Glycine. Downstream product: Chlorophyll a/b, pyropheophorbide a.
		Pheophorbide a	0.295	Down	An intermediate in chlorophyll degradation. Upstream product: Chlorophyll a/b. Downstream product: Pyropheophorbide a, chlorophyll catabolite.
		Pyropheophorbide a	0.157	Down	One of a final products in chlorophyll degradation. Upstream product: Pheophorbide a.

## Data Availability

The original contributions presented in this study are included in the article/Supplementary Material. Further inquiries can be directed to the corresponding author.

## Supplementary Materials

The following supporting information can be downloaded at: https://www.mdpi.com/article/10.3390/microorganisms14030561/s1.
