# Supplementary material for: Multiomics Approach Reveals the Inhibitory Effects of Protocatechuic Acid on the Marine Dinoflagellate Scrippsiella acuminata
Source: Microorganisms. 2026 Mar 1;14(3):561. doi: 10.3390/microorganisms14030561 (PMC13028991; doi:10.3390/microorganisms14030561)
Supplement: Supplementary file 1 [file microorganisms-14-00561-s001.zip › Supplementary materials S1. Supplementary figures and tables.pdf]

Multiomics Approach Reveals the Inhibitory Effects of Protocatechuic Acid on the Marine Dinoflagellate *Scrippsiella acuminata*

Xin Zhang[1, †], Mei-yao He[2, †], Di Wang[1], Meimei Wang[1], Hongxin Liu[1], Jihui Wang[1], Shunshan Duan[2] and Meng Liu[1, *]

1 School of Life and Health Technology, Dongguan University of Technology, Dongguan 523808, China;

2 Department of Ecology, Jinan University, Guangzhou 510632, China.

* Corresponding author e-mail address: liumengpro2015@outlook.com (M. Liu)

† These authors contributed equally to this work.

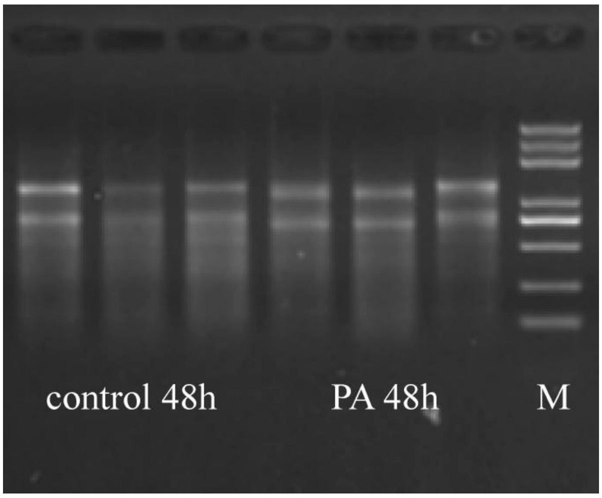

Figure S1. Electrophoresis of total RNA

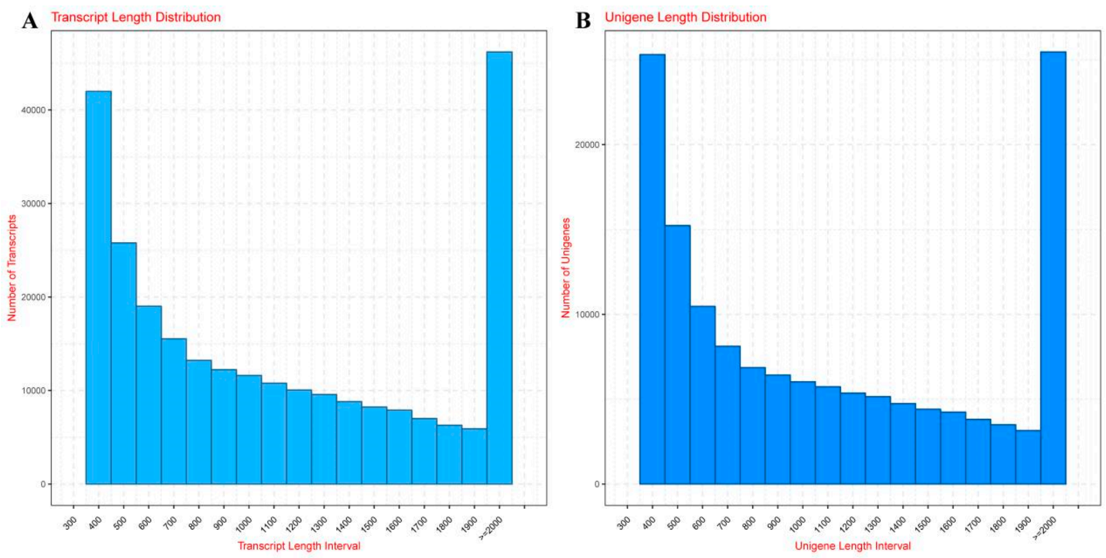

Figure S2. Length distributions of transcript(A) and unigene(B)

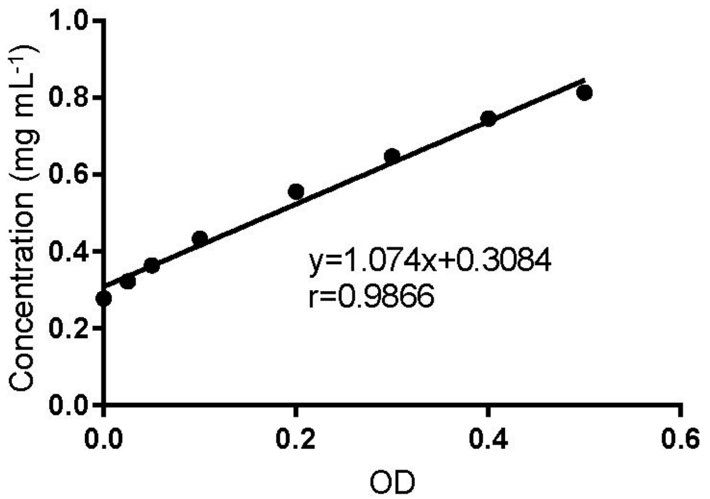

Figure S3. Bradford protein quantitative standard curve.

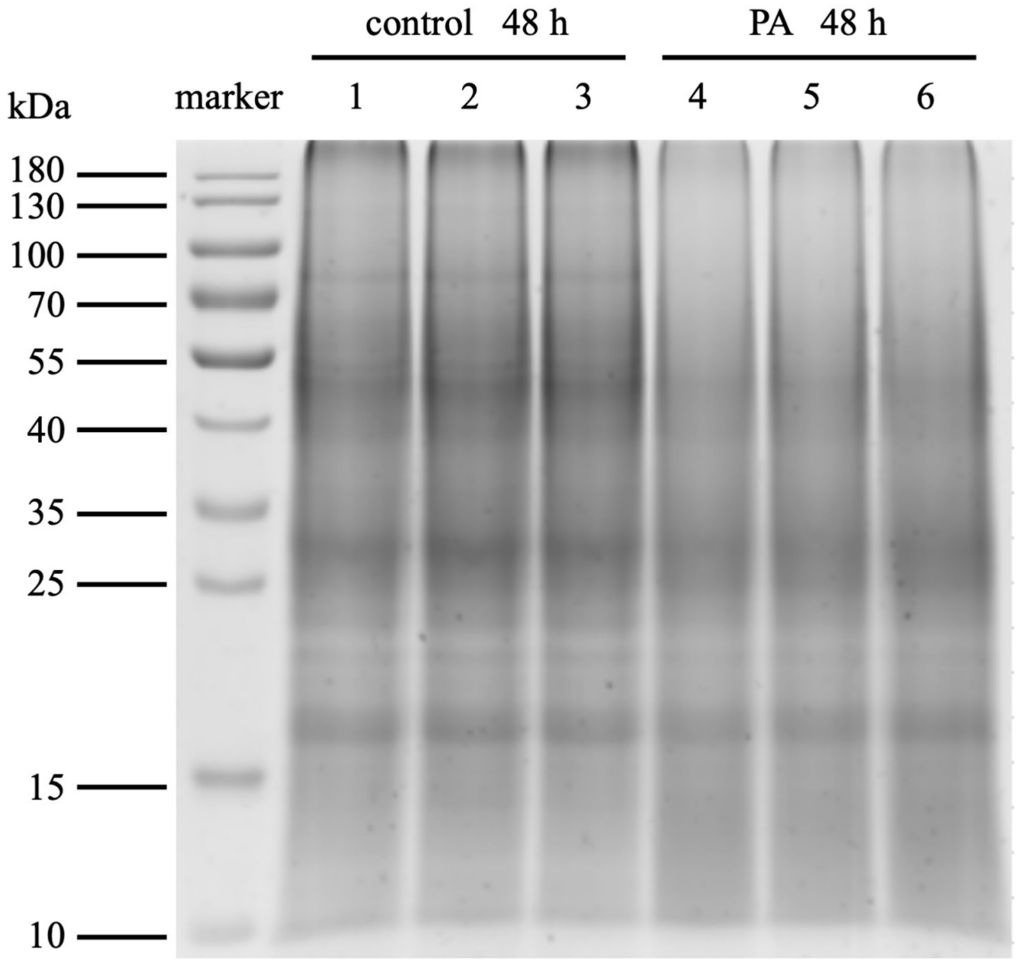

Figure S4. SDS-PAGE electrophoresis detection. 1-3: control group. 4-6: treatment group. Marker stands for 180, 130, 100, 70, 55, 40, 35, 25, 15 and 10 kDa from top to bottom.

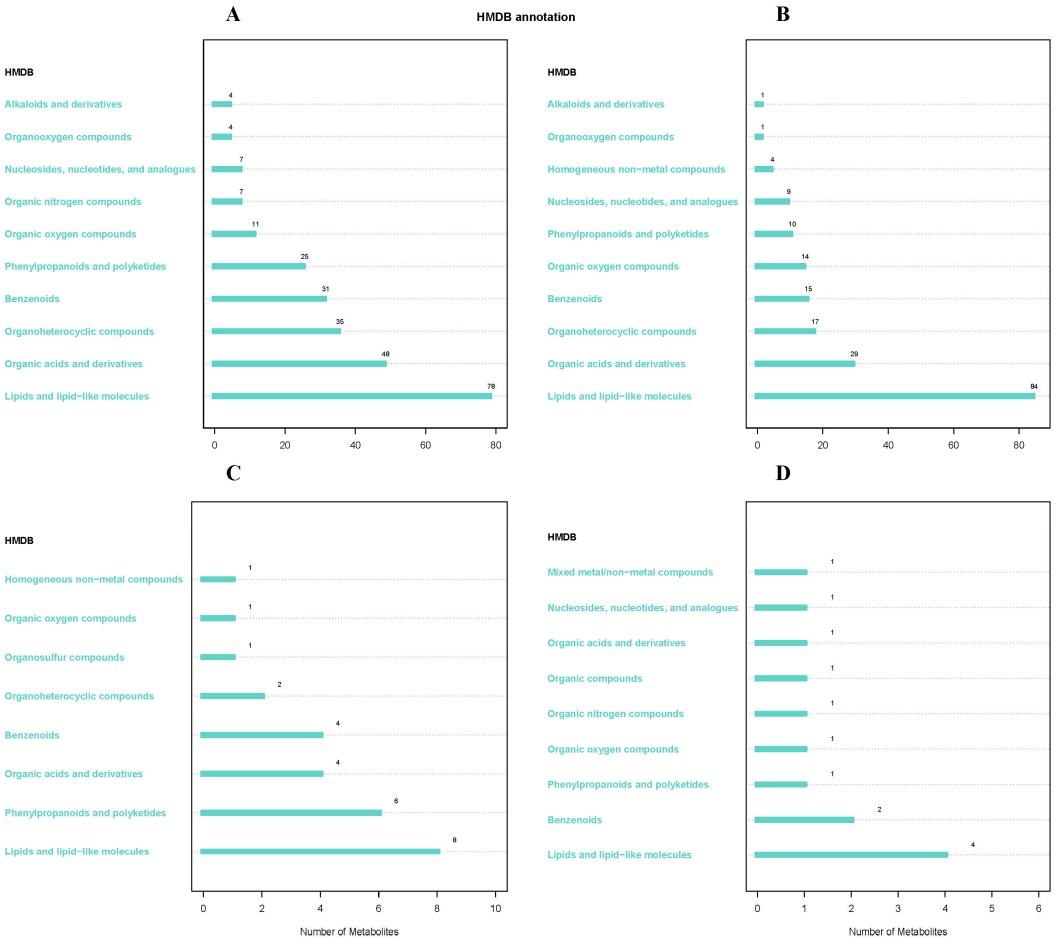

Figure S5. KEGG annotation of intracellular (A, B) and extracellular (C, D) metabolites under positive (A, C) and negative (B, D) ion modes.

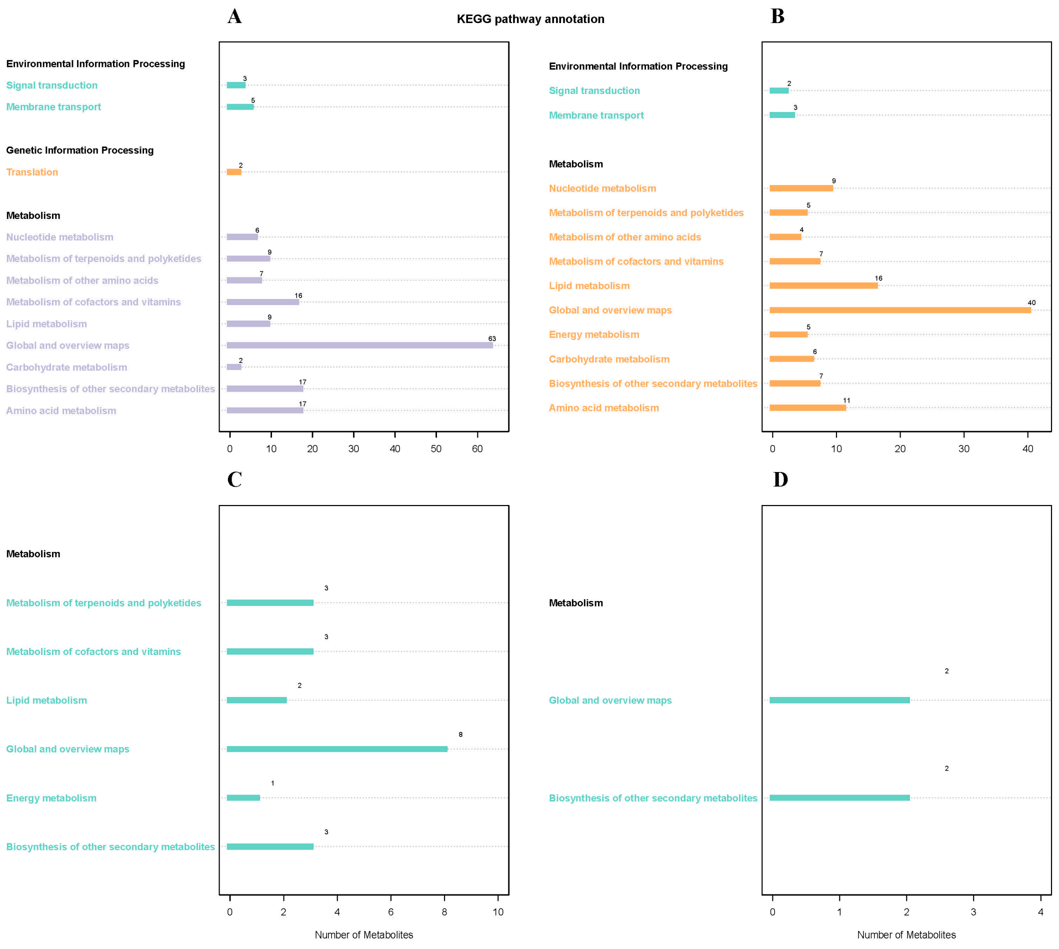

Figure S6. HMBD annotation of intracellular (A, B) and extracellular (C, D) metabolites under positive (A, C) and negative (B, D) ion modes.

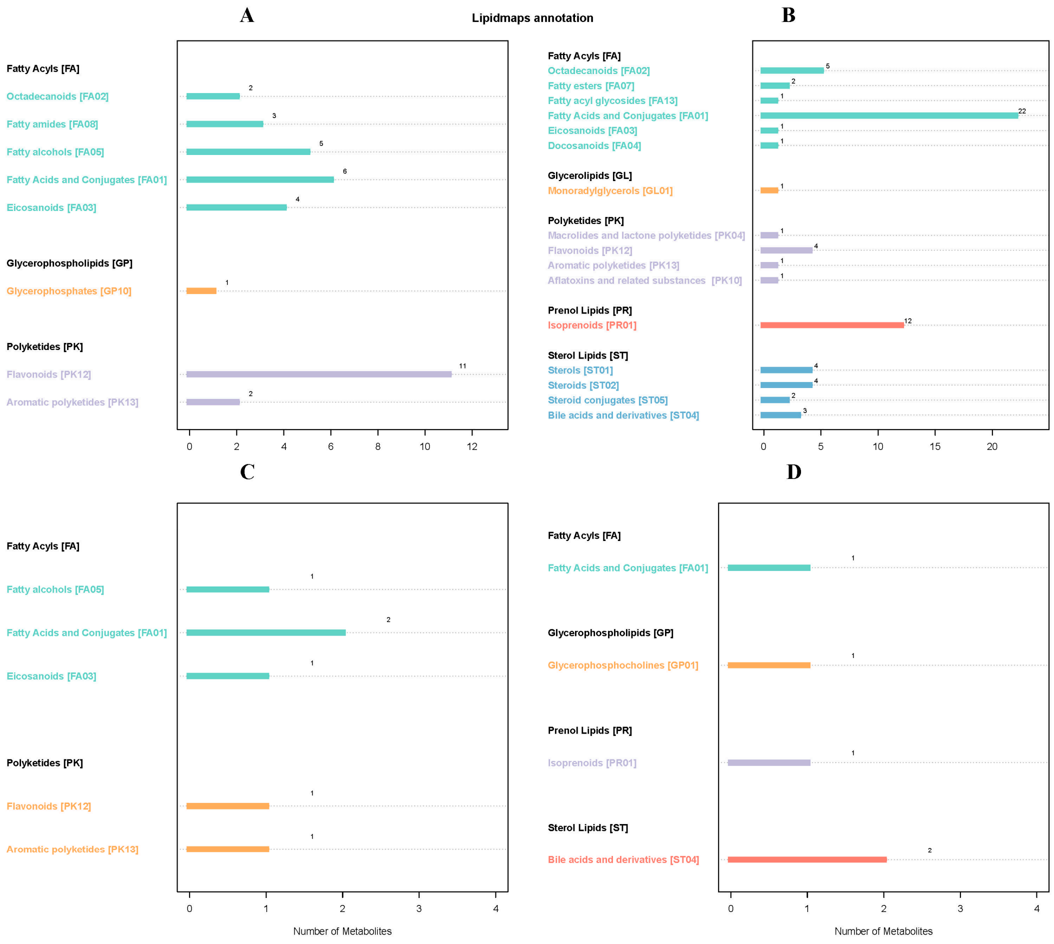

Figure S7. LIPID MAPS annotation of intracellular (A, B) and extracellular (C, D) metabolites under positive (A, C) and negative (B, D) ion modes.

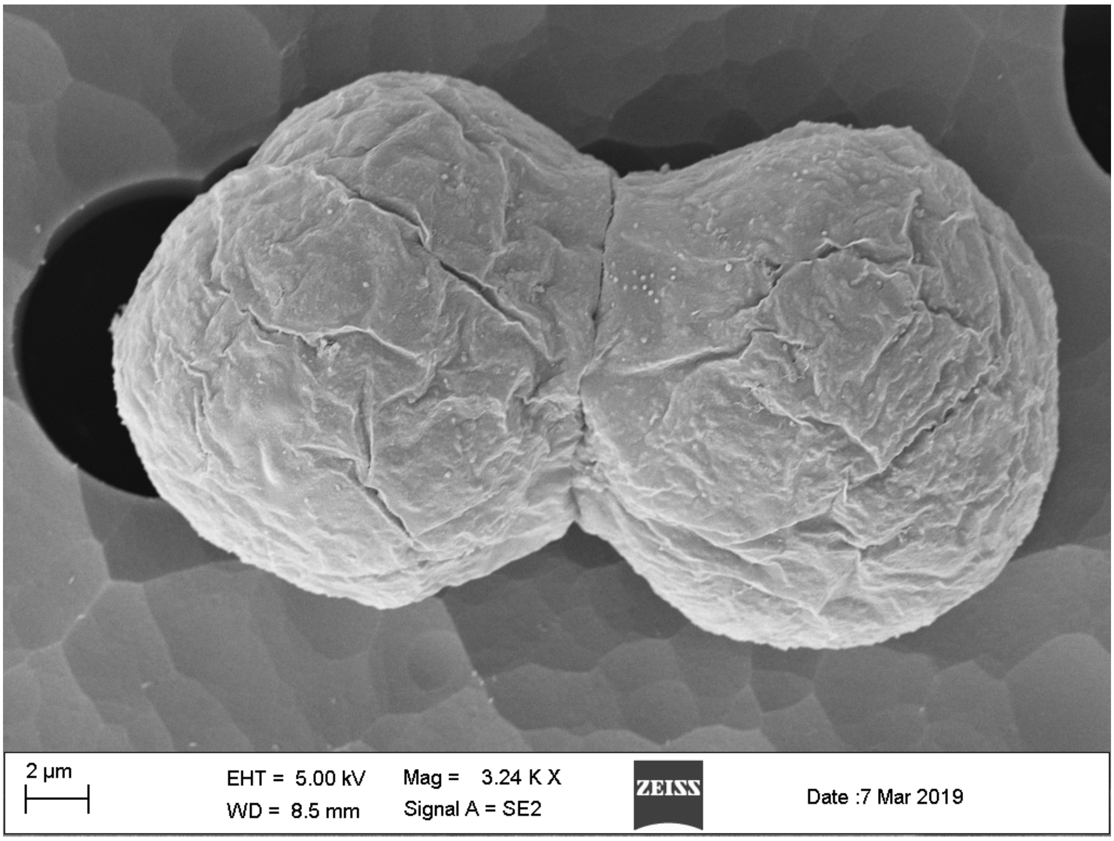

Figure S8. The photo of Resting cyst formation by scanning electron microscope

Table S1 Quality of sample total RNA.

Sample-ID	Concentration (ng/ul)	Content (ug)	OD260/280	RIN
control 48h-1	312	9.984	2.36	6.80
control 48h-2	228	7.296	2.16	5.20
control 48h-3	186	5.952	2.02	5.70
PA 48h-4	148	4.736	2.18	6.40
PA 48h-5	90	2.88	2.22	5.70
PA 48h-6	246	7.872	2.32	6.10

Table S2 Summary of sequencing data.

Sample	Clean reads	Clean bases	Error %	Q20 %	Q30 %	GC %
control 48h-1	98224690	14.73G	0.02	98.35	95.33	62.15
control 48h-2	69952386	10.49G	0.02	98.48	95.69	62.28
control 48h-3	74348196	11.15G	0.02	98.39	95.45	62.04
PA 48h-1	84376120	12.66G	0.02	98.4	95.42	61.65
PA 48h-2	103232972	15.48G	0.02	98.23	95.08	61.84
PA 48h-3	87938152	13.19G	0.02	98.41	95.43	62.28

Table S3 Annotation results of DEGs (PA 48 h vs. control 48 h).

DEGs	Annotated	Nr	Nt	KO	SwissProt	PFAM	GO	KOG
5328	4553	3836	931	2023	3471	3734	3734	2534

Table S4 List of top20 up- and down-regulated in KEGG pathway enrichment of DEGs (PA 48h vs control 48h).

Metabolic pathway	Amount	Up/Down	Annotation of DEGs	pajd
Proteasome	58	Up	POMP, PSMD3, PSMD12, PSMD11, PMSD6, PSMD7, PSMD13, PSMD14, PSMD4, PSMD2, PSMD1, PSMD13, PSMC2, PSMC1, PSMC5, PSMC6 , PSMC3, PSMC4, PSMA6, PSMA2, PSMA3, PSMA4, PSMA5, PSMA6, PSMA7, PSMB6, PSMB7, PSMB3, PSMB2, PSMB5, PSMB1, PSMB4	2.61E-10
Ribosome	195	Up	RP-L3, RP-S20e, RP-L3e, RP-L4e, RP-L23Ae, RP-L8e, RP-S15e, RP-L22, RP-L17e, RP-S3e, RP-L35e, RP-S17, RP-L24, RP-S4e, RP-L26e, RP-L23e, RP-S11e, RP-L11e, RP-S29e, RP-S15Ae, RP-L9e, RP-L32e, RP-L19e, RP-L5e, RP-S2e, RP-L7e, RP-L27Ae, RP-L34e, RP-L14e, RP-S18e, RP-S14e, RP-S9e, RP-L18e, RP-L17, RP-L13, RP-L13Ae, RP-S9, RP-S16e, RP-SAe, RP-S13e, RP-L20, RP-L28, RP-L21, RP-L27, RP-L10e, RP-L13e, RP-L15e, RP-L21e, RP-L24e, RP-L31e, RP-L35Ae, RP-L37e, RP-L37Ae, RP-L40e, RP-L44e, RP-S3Ae, RP-S6e, RP-S8e, RP-S17e, RP-S19e, RP-S24e, RP-S25e, RP-S26e, RP-S27e, RP-S27Ae, RP-L6e, RP-L18Ae, RP-L22e,	3.71E-07

			RP-L27e, RP-L28e, RP-L36e, RP-L38e, RP-S7e, RP-S10e, RP-S12e, RP-S21e	
Steroid biosynthesis	19	Up	FDFT1, ERG1, CYP51, ERG24, ERG3, DHCR7, LIPA, ERG6, ERG4, CAS1, CPI1, CYP51, CYP710A	0.016105214
Oxidative phosphorylation	53	Up	NDUFS4, NDUFS6, NDUFS7, NDUFS8, NDUFV1, NDUFV2, NDUFA5, NDUFA9, NDUFAB1, NDUFA12, NDUFB9, SDHC, SDHD, SDHA, SDHB, UQCRFS1, fbcH, QCR6, COX10, COX5B, COX6B, COX15, ATPeF1A, ATPeF1B, ATPeF1G, ATPeF1D, ATPeF0O, ATPeV1A, ATPeV1B, ATPeV1E, ATPeV1F, ATPeV1G, ATPeV0A, ATPeV0C, ATPeV0D	0.022756169
Ribosome biogenesis in eukaryotes	40	Up	CSNK2A, UTP22, UTP18, PWP2, MPP10, NOP1, NOP56, NOP58, SNU13, DKC1, NHP2, GAR1, NOP10, UTP14, EMG1, NAT10, RCL1, NOG1, NUG2, REX1, RIX7, XRN1, NOB1, RAN, XPO1, NMD3, RIOK1, EIF6, SDO1, RIA1, LSG1	0.046408785
Phagosome	47	Up	NDUFS4, NDUFS6, NDUFS7, NDUFS8, NDUFV1, NDUFV2, NDUFA5, NDUFA9, NDUFAB1, NDUFA12, NDUFB9, SDHC, SDHD, SDHA, SDHB, UQCRFS1, fbcH, QCR6, COX10, COX5B, COX6B, COX15, ATPeF1A, ATPeF1B, ATPeF1G, ATPeF1D, ATPeF0O, ATPeV1A, ATPeV1B, ATPeV1E, ATPeV1F, ATPeV1G, ATPeV0A, ATPeV0C, ATPeV0D	0.141821933
N-Glycan biosynthesis	21	Up	ALG7, ALG11, DPM1, ALG3, ALG9, ALG12, ALG5, STT3, RPN1, MOGS, GANAB, MAN1A_C, MGAT1	0.141821933
Fatty acid elongation	9	Up	MECR, PPT, HSD17B12, HACD, TER	0.141821933
Valine, leucine and isoleucine degradation	34	Up	ilvE, BCKDHA, DLD, DBT, IVD, MCCC1m, HMGCS, ACAT, HIBADH, fadA, AGXT2, ALDH, ACSF3, PCCA, MUT, MCEE	0.180251264
Citrate cycle (TCA cycle)	43	Up	pckA, MDH1, fumA, SDHA, LSC1, sucD, DLST, DLD, OGDH, IDH3, ACO, ACLY, CS, DLAT, aceE, PC	0.286481641
Biosynthesis of unsaturated fatty acids	15	Up	ACOX1, ACAA1, HSD17B12, HACD, fabG, FAD2, SCD, FADS2, TER	0.314467137
Aminoacyl-tRNA biosynthesis	32	Up	EARS, QARS, AARS, NARS, TARS, SARS, CARS, MARS, VARS, LARS, IARS, lysK, PARS, HARS, FARSA, YARS, WARS	0.488869932
Arachidonic acid metabolism	12	Up	CBR1, PTGES, PTGDS, LTA4H, GGT1_5, gpx	0.488869932
Ubiquitin mediated proteolysis	50	Up	UBE1, UBA3, UBE2A, UBE2I, UBE2C, UBE2S, UBE2D, UBE2E, UBE2W, UBE2M, UBE2G1, UBE2N, UBE2O, UBE2H, TRIP12, HUWE1, HERC4, PRPF19, PIAS1, SYVN1, RBX1, CUL1, SKP1, GRR1, APC2, CDC20, APC10, ELOC, CUL3, CUL4, ELOC, SKP1	0.754793292
Lysine degradation	12	Up	AASS, ALDH7A1, OGDH, DLST, ACAT, ALDH	0.766902078

Amino sugar and nucleotide sugar metabolism	22	Up	nagZ, glmS, GPI, GLCAK, pgm, GMPP, GME, galE, UGP2, UGDH, UXS1, CYB5R, RHM, UER1,	0.766902078
Arginine and proline metabolism	21	Up	aguA, ODC1, rocD, proA, proB, E1.2.1.88, PRODH, pip, P4HA, GOT1, speD, ALDH,	0.768601689
Glycosphingolipid biosynthesis - globo series	6	Up	GLA, HEXA_B	0.999999561
Glycosphingolipid biosynthesis - ganglio series	4	Up	HEXA_B	0.999999561
Tryptophan metabolism	12	Up	ALDH, AAO1_2, KMO, CAT, ACAT, OGDH	0.999999561
Photosynthesis	3	Down	psbB, psaA, psaB	7.45E-05
Riboflavin metabolism	1	Down	ribB, ribA	0.020638422
Nitrogen metabolism	1	Down	GLU	0.047930975
Glyoxylate and dicarboxylate metabolism	1	Down	GLU	0.099264963

Table S5. DEPs functional description and Related metabolic pathway.

Protein	Fold change	Related metabolic pathway
**Lipid & Sterol Metabolism**		
Sterol 24-C-methyltransferase	1.55	Phytosterol biosynthesis
Cytochrome b5	1.77	Endoplasmic-reticulum electron transport
Acyl-CoA dehydrogenase	1.88	Fatty-acid β-oxidation
Acyl-CoA dehydrogenase	1.78	Fatty-acid β-oxidation
Sterol-4-alpha-carboxylate 3-dehydrogenase	1.85	Post-squalene sterol biosynthesis
Acetyl-CoA acetyltransferase	1.51	Ketone-body / Isoprenoid synthesis
Phospholipid N-methyltransferase	1.66	Phosphatidylcholine biosynthesis
Lipoprotein metabolic process	1.57	Lipoprotein metabolism
Acyltransferase family protein	1.88	Lipid metabolism
**Carbohydrate Metabolism**		
Xylan 1,4-beta-xylosidase	1.73	Hemicellulose degradation
Granule-bound starch synthase 1	1.53	Starch biosynthesis (amylose)
Granule-bound starch synthase 1	2.05	Starch biosynthesis (amylose)
putative beta-1,3-galactosyltransferase 20	1.64	Protein glycosylation / Cell-wall biosynthesis
**Glycolysis**		
6-phosphofructokinase	1.76	Glycolysis
**TCA Cycle & Anaplerosis**		
Malate dehydrogenase	1.54	Citrate cycle (TCA cycle); Cysteine and methionine metabolism; Pyruvate metabolism; Glyoxylate and dicarboxylate metabolism; Carbon fixation in photosynthetic organisms
Oxaloacetate decarboxylase, gamma chain	2.57	Anaplerotic/CO_2-concentrating mechanism
Fumarate reductase	1.59	Citrate cycle (TCA cycle); Pyruvate metabolism; Butanoate metabolism
**Nitrogen & Nitrate Assimilation**		
Nitrate transporter	1.74	Nitrate uptake / Nitrogen assimilation
Glutamate synthase 1 [NADH]	1.70	Alanine, aspartate and glutamate metabolism; Nitrogen metabolism
NAD(P)H-nitrite reductase, large subunit	1.55	Nitrogen assimilation
**Respiratory Electron Transport & Ubiquinone Synthesis**		
Ubiquinone/menaquinone biosynthesis C-methylase	1.65	Ubiquinone / Menaquinone biosynthesis

UbiE		
Cytochrome c	1.65	Mitochondrial electron-transport chain

Redox Detoxification & Glutathione System

Glutathione S-transferase	1.68	Detoxification / Redox homeostasis
Glutathione S-transferase	1.52	Detoxification / Redox homeostasis
Glutamate--cysteine ligase catalytic subunit	2.06	Cysteine and methionine metabolism; Glutathione metabolism
Glutathionyl-hydroquinone reductase	1.88	Chloroplast redox homeostasis
Glutathione-independent glyoxalase hsp3101	1.59	Glyoxalase system / Stress response

Photosynthetic & Ferredoxin Electron Transport

Ferredoxin	2.68	Photosynthetic electron transport
Ferredoxin	1.79	Photosynthetic electron transport

Secondary Metabolism / Detoxification

Cytochrome P450	2.15	Secondary metabolism / Detoxification
Dehydrogenase [NADP..+..]	2.36	Energy production and conversion
dehydrogenase [NADP..+..]	2.40	Energy production and conversion

Coenzyme A & Pantothenate Biosynthesis

3-methyl-2-oxobutanoate hydroxymethyltransferase	1.51	Pantothenate (CoA) biosynthesis

Nucleotide Metabolism

Nucleoside diphosphate kinase	1.73	Nucleotide interconversion

Protein Homeostasis & Folding

Chaperone protein ClpC1, chloroplastic	5.50	Chloroplast protein quality control

Signal Transduction

RasGAP-activating-like protein 1	1.67	Signal transduction
Calmodulin	1.99	Calcium signaling
sodium bicarbonate cotransporter 1	1.63	pH regulation / CO_2 concentrating mechanism

DNA Replication & Central Dogma

Proliferating cell nuclear antigen	1.56	DNA replication; Base excision repair; Nucleotide excision repair; Mismatch repair
Ribosomal protein S6	1.87	Ribosome
RNA polymerase Rpb3/Rpb11 dimerization domain	1.51	transcription, DNA-templated

Photosynthetic Electron Transport & Light-Harvesting

light-harvesting complex I chlorophyll a/b binding protein 1	0.64	Photosynthetic light reactions

light-harvesting complex I chlorophyll a/b binding protein 1	0.67	Photosynthetic light reactions
Fucoxanthin-chlorophyll a-c binding protein F, chloroplastic	0.63	Photosynthetic light reactions
photosystem II oxygen-evolving enhancer protein 3	0.52	Photosystem II water-splitting complex

Secondary Metabolism & Detoxification

beta-glucosidase	0.60	Cell-wall catabolism / Secondary metabolism
Cytochrome P450 CYP72A219	0.63	Secondary metabolism / Detoxification
O-methyltransferase MdmC	0.63	Secondary metabolism

Chromatin Remodeling & Epigenetic Regulation

tetratricopeptide repeat protein 38	0.65	Nuclear RNA-protein interactions
Lysine-specific demethylase 8	0.65	Epigenetic regulation

DNA Repair

Alkylated DNA repair dioxygenase AlkB	0.59	Base-excision repair

Ribosome Assembly & Translation

large subunit ribosomal protein L40e	0.55	Ribosome (60S subunit)
Ribosomal protein L14E/L6E/L27E/60S ribosomal protein L14	0.64	Ribosome (60S subunit)
Ribosomal L29e protein family	0.64	Ribosome (60S subunit)
U3 small nucleolar RNA-associated protein 14	0.63	Nucleolar rRNA processing
rRNA methyltransferase activity	0.66	rRNA modification (nucleolus)
30S ribosomal protein subunit S22 family	0.60	Organellar ribosome (30S subunit)

Coenzyme A Biosynthesis

dephospho-CoA kinase	0.61	Coenzyme A biosynthesis

TCA Cycle & Mitochondrial Energy Conversion

Malate dehydrogenase, mitochondrial	0.65	Mitochondrial TCA cycle

Flagellar / Ciliary Energy Metabolism

Creatine kinase, flagellar	0.640	Ciliary energy metabolism

Signal Transduction

3',5'-cyclic AMP phosphodiesterase CpdA	0.53	cAMP signaling pathway
cAMP-dependent protein kinase regulator	0.65	cAMP signaling pathway
calcium-dependent protein kinase	0.60	Calcium signaling pathway
3-phosphoinositide-dependent protein kinase 2	0.51	PI3K–AKT signaling pathway
Calcium-activated potassium channel, beta subunit	0.60	Ion-channel regulation
Zinc finger, ZZ type	0.67	Ubiquitin–proteasome system / Signal transduction

**Protein Folding & Chaperone Activity**		
ATP-dependent carboligase	0.62	Secondary metabolism
**Protein Degradation**		
cathepsin H	0.63	Lysosomal proteolysis
**Protein/Organelle Trafficking & Positioning**		
LysE type translocator	0.55	Membrane transport / Secretion
centrosomal protein CEP104	0.65	Centrosome / Ciliogenesis
**Transcription / RNA Processing**		
Zinc finger C-x8-C-x5-C-x3-H type	0.66	Nuclear regulatory complexes
**Apoptosis / p53-Mediated Cell Death Pathway**		
death-inducing p53-target protein 1-like	0.66	p53-mediated apoptosis

Table S6. DEMs in *Scrippsiella acuminata* in HMBD and Lipidmaps (PA 48h vs control 48h).

Database	Category (amount)	Upregulated metabolites (chemical formula)	Downregulated metabolites (chemical formula)
HMBD	Nucleosides, nucleotides, and analogues (2)	deoxyuridine ($C_9 H_{12} N_2 O_5$)<br>dUDP ($C_9 H_{14} N_2 O_{11} P_2$)	-
	Benzenoids (4)	-	3-Methoxybenzaldehyde ($C_8 H_8 O_2$)<br>Hydroquinone ($C_6 H_6 O_2$)<br>Butylated hydroxytoluene ($C_{15} H_{24} O$)<br>anacardic acid ($C_{22} H_{36} O_3$)
	Lipids and lipid-like molecules (31)	13-KODE ($C_{18} H_{30} O_3$)<br>coenzyme Q2 ($C_{19} H_{26} O_4$)<br>Lubiprostone ($C_{20} H_{32} F_2 O_5$)<br>Thiothixene ($C_{23} H_{29} N_3 O_2 S_2$)<br>Hendecanoic Acid ($C_{17} H_{32} O_2$)<br>Nomilin ($C_{28} H_{34} O_9$)<br>oxandrolone ($C_{19} H_{30} O_3$)<br>hydroxyprogesterone caproate ($C_{27} H_{40} O_4$)<br>Ziziphin ($C_{51} H_{80} O_{18}$)<br>Jujuboside B ($C_{52} H_{84} O_{21}$)<br>PHYLLOQUINONE OXIDE ($C_{31} H_{46} O_3$)<br>cholic acid ($C_{24} H_{40} O_5$)<br>Tetrahydrodeoxycorticosterone ($C_{21} H_{34} O_3$)<br>Araloside A ($C_{47} H_{74} O_{18}$)	Nandrolone ($C_{18} H_{26} O_2$)<br>Dihomo-gamma-linolenic acid ($C_{20} H_{34} O_2$)<br>Hydrocortisone Valerate ($C_{26} H_{38} O_6$)<br>Budesonide ($C_{25} H_{34} O_6$)<br>Astaxanthin ($C_{40} H_{52} O_4$)<br>Latanoprost ($C_{26} H_{40} O_5$)<br>Geranylacetone ($C_{13} H_{22} O$)<br>L-alpha-Glycerylphosphorylcholine ($C_8 H_{20} N O_6 P$)<br>LysoPC(18:3(9Z,12Z,15Z)) ($C_{26} H_{48} N O_7 P$)<br>soyasapogenol A ($C_{30} H_{50} O_4$)<br>Garcinol ($C_{38} H_{50} O_6$)

		Medicagenic acid 3-O-triglucoside (C48 H76 O21)<br>Corticosterone (C21 H30 O4)<br>Methyl Jasmonate (C13 H20 O3)<br>Oseltamivir (C16 H28 N2 O4)<br>13S-hydroxyoctadecadienoic acid (C18 H32 O3)	Estriol (C18 H24 O3)
	Organic acids and derivatives (18)	Bradykinin (C50 H73 N15 O11)<br>carnosine (C9 H14 N4 O3)<br>DL-2,6-Diaminopimelic acid (C7 H14 N2 O4)<br>N-Acetylornithine (C7 H14 N2 O3)<br>ala-met (C8 H16 N2 O3 S)<br>Acevaltrate (C24 H32 O10)<br>Leucylproline (C11 H20 N2 O3)<br>L-Asparaginyl-L-threonine (C8 H15 N3 O5)<br>ser-gly (C5 H10 N2 O4)<br>Asp-lys (C10 H19 N3 O5)<br>Gly-Leu (C8 H16 N2 O3)<br>asp-arg (C10 H19 N5 O5)<br>L-Theanine (C7 H14 N2 O3)<br>Kyotorphin (C15 H23 N5 O4)<br>Ala-Leu (C9 H18 N2 O3)	Angiotensin II (C50 H71 N13 O12)<br>12-Hydroxylauric acid (C12 H24 O3)<br>Tiagabine (C20 H25 N O2 S2)
	Organic oxygen compounds (2)	2,3-Diphosphoglyceric acid (C3 H8 O10 P2)	Lauryl aldehyde (C12 H24 O)
	Organoheterocyclic compounds (8)	Paliperidone (C23 H27 F N4 O3)<br>Dihydrothymine (C5 H8 N2 O2)<br>Adenine (C5 H5 N5)<br>Protoporphyrin IX (C34 H34 N4 O4)<br>(+)-Simvastatin (C25 H38 O5)	Riboflavin Tetrabutyrate (C33 H44 N4 O10)<br>Desloratadine (C19 H19 Cl N2)<br>Olomoucine (C15 H18 N6 O)
	Organic nitrogen compounds (1)	-	bis(3-aminopropyl)amine (C6 H17 N3)
	Organooxygen compounds (2)	-	(+/-)-Muscone (C16 H30 O)<br>Dihydrojasmone (C11 H18 O)
	Phenylpropanoids and polyketides (3)	flavoxate (C24 H25 N O4)<br>Gingerenone A (C21 H24 O5)<br>6,7,8-Trihydroxy-2H-chromen-2-one (C9 H6 O5)	rolitetracycline (C27 H33 N3 O8)<br>Loxoprofen (C15 H18 O3)
Lipidmaps	Fatty Acyls (8)	2-Amino-9,10-epoxy-8-oxodecanoic acid (C10 H17 N O4)	10-Undecenoic acid (C11 H20 O2)

	13-KODE (C18 H30 O3)<br>13-Tetradecynoic acid (C14 H24 O2)<br>13-(beta-D-glucosyloxy)docosanoic acid (C28 H54 O8)<br>Acevaltrate (C24 H32 O10)<br>pre-putrebactin (C16 H30 N4 O7)<br>Deferoxamine (C25 H48 N6 O8)<br>Methyl Jasmonate (C13 H20 O3)	
Polyketides (4)	amorphigenin (C23 H22 O7)<br>Avermectin B1b (C47 H70 O14)	millettone (C22 H18 O6)<br>anacardic acid (C22 H36 O3)
Prenol Lipids (4)	Loganin (C17 H26 O10)<br>Harpagoside (C24 H30 O11)	Astaxanthin (C40 H52 O4)<br>Bayogenin 3-O-cellobioside (C42 H68 O15)
Sterol Lipids (6)	cholic acid (C24 H40 O5)<br>Tetrahydrodeoxycorticosterone (C21 H34 O3)<br>doxercalciferol (C28 H44 O2)<br>Corticosterone (C21 H30 O4)<br>Diosgenin (C27 H42 O3)	Nandrolone (C18 H26 O2)

Table S7. Abbreviation List

Abbreviation	Full name
19S	19S Proteasome Regulatory Particle
2.3.3.10	3-Hydroxy-3-methylglutaryl-CoA Synthase
ACACA	Acetyl-CoA Carboxylase Alpha
ACAT	Acetyl-CoA Acetyltransferase
acd	Acyl-CoA Dehydrogenase
ACLY	ATP Citrate Lyase
ACO	Aconitase
ACOX	Acyl-CoA Oxidase
ACSL	Acyl-CoA Synthetase Long-Chain Family Member
ALKBH1	AlkB Homolog 1, DNA Repair Protein
ATG4	Autophagy-Related 4 Cysteine Protease
atpB	ATP Synthase Subunit Beta
bglB	Beta-Glucosidase
CALM	Calmodulin

CAS1	Carboxylesterase 1
CAT	Catalase
ccoN	Cytochrome C Oxidase Cbb3-Type Subunit I
COX10	Cytochrome C Oxidase Assembly Factor
COX15	Cytochrome C Oxidase Assembly Protein
cpdA	3',5'-Cyclic Nucleotide Phosphodiesterase
CPI1	Cyclopentanol Dehydrogenase
CPK	Calcium-Dependent Protein Kinase
CS	Citrate Synthase
CYP51	Sterol 14-Alpha-Demethylase
DamX	DamX Protein
DHCR7	7-Dehydrocholesterol Reductase
DLAT	Dihydrolipoyllysine Acetyltransferase
E1	Ubiquitin-Activating Enzyme E1
E2	Ubiquitin-Conjugating Enzyme E2
E3	Ubiquitin Ligase
EARS	Glutamyl-tRNA Synthetase
EMG1	rRNA Small Subunit Pseudouridine Methyltransferase Nep1
ENO	Enolase
ERG24	24-Demethylase
ERG3	5-Desaturase
ERG4	24-Demethylase
ERG6	Sterol 24-C-Methyltransferase
FabG	3-Ketoacyl-ACP Reductase
fadA	Acyl-CoA Synthetase
FBA	Fructose-Bisphosphate Aldolase
fbcH	Ubiquinol-Cytochrome C Reductase Iron-Sulfur Subunit
FBP	Fructose-1,6-Bisphosphatase
FDFT1	Farnesyl Diphosphate Farnesyltransferase

FDPS	Farnesyl Diphosphate Synthase
Fum	Fumarase
GADPH	Glyceraldehyde-3-Phosphate Dehydrogenase (NADP+)
GCLC	Glutamate-Cysteine Ligase Catalytic Subunit
GDH	Glutamate Dehydrogenase
GGT	Gamma-Glutamyltranspeptidase
glgA	Glycogen Synthase
glnA	Glutamine Synthetase
GLT1	Glutamate Synthase (NADH)
glts	Glutamate Synthase (Ferredoxin)
GPI	Glucose-6-Phosphate Isomerase
gpx	Glutathione Peroxidase
GSR	Glutathione Reductase (NADPH)
GST	Glutathione S-Transferase
hemB	Delta-Aminolevulinate Synthase
hemE	Uroporphyrinogen Decarboxylase
HMGCR	3-Hydroxy-3-Methylglutaryl-CoA Reductase
HSP100	Heat Shock Protein 100
HSP31	Lactoylglutathione Lyase
IDH3	Isocitrate Dehydrogenase (NAD+)
KDM8	Lysine-Specific Demethylase 8
KRE33	N-Acetyltransferase 10
LCHA1	Light-Harvesting Complex I Chlorophyll A/B-Binding Protein 1
LITAF	Lipopolysaccharide-Induced TNF-Alpha Factor
LSC	Succinyl-CoA Synthetase Subunit
lysE	Lysine Exporter Family Protein
mdh	Malate Dehydrogenase
MPV17	Mpv17 Protein
mvaD	Dimethylallyl Diphosphate Synthase

ND1	NADH-Ubiquinone Oxidoreductase Subunit
ndh	NADH Dehydrogenase
ndk	Nucleoside Diphosphate Kinase
NHP	H/ACA Ribonucleoprotein Complex Subunit 2
nirB	Nitrite Reductase (NADH) Large Subunit
NMD3	Nonsense-Mediated MRNA Decay Protein 3
NOLA	H/ACA Ribonucleoprotein Complex Subunit 3
NOP	Ribonuclease P/MRP Subunit P
NR	Nitrate/Nitrite Transporter
NRT	Nitrate/Nitrite Transporter
NSDHL	Sterol-4-alpha-Carboxylate 3-Dehydrogenase (Decarboxylating)
nuoA	NADH-Ubiquinone Oxidoreductase Subunit A
oadG	Oxaloacetate Decarboxylase Subunit
OGDH	2-Oxoglutarate Dehydrogenase E1 Component
Omtf	Adenosyl-L-Methionine Dependent Methyltransferase Superfamily Protein
PAC4	Proteasome Assembly Chaperone 4
PCNA	Proliferating Cell Nuclear Antigen
PDHA	Pyruvate Dehydrogenase E1 Component Alpha Subunit
PDPK1	3-Phosphoinositide-Dependent Protein Kinase-1
PEMT	Phosphatidylethanolamine N-Methyltransferase
PEX2	Peroxisomal Biogenesis Factor 2
PEX5	Peroxisomal Biogenesis Factor 5
PEX6	Peroxisomal Biogenesis Factor 6
PFP	Fructose-Bisphosphate Aldolase
PGAM	Phosphoglycerate Mutase 2,3-Bisphosphoglycerate-Dependent
PGC	Gastricsin
PGK	Phosphoglycerate Kinase
PGM	Phosphoglucomutase

PIT	Calcium-Activated Potassium Channel Subunit
PKLR	Pyruvate Kinase L/R
PMA	H+-Transporting ATPase
PMP70	ATP-Binding Cassette, Subfamily D (ALD), Member 3
porA	Proton-Translocating NADH-Quinone Oxidoreductase Subunit
ppa	Inorganic Pyrophosphatase
PsaA	Photosystem I P700 Chlorophyll A Apoprotein A1
PsaB	Photosystem I P700 Chlorophyll A Apoprotein A2
PsbB	Photosystem II CP47 Chlorophyll Apoprotein
psbQ	Photosystem II Oxygen-Evolving Enhancer Protein 3
QCR2	Ubiquinol-Cytochrome C Reductase Core Subunit 2
Rad50	DNA Repair Protein RAD50
RAN	GTP-Binding Nuclear Protein Ran
RASA1	Ras GTPase-Activating Protein 1
Rcl1	RNA 3'-Terminal Phosphate Cyclase
ripA	Ribose- 5-Phosphate Isomerase A
RPE	Ribulose-Phosphate 3-Epimerase
RPL14	60S Ribosomal Protein L14
RPL40	60S Ribosomal Protein L40
RPS6	40S Ribosomal Protein S6
SDH	Succinate Dehydrogenase (Ubiquinone) Flavoprotein Subunit
SDHA	Succinate Dehydrogenase (Ubiquinone) Flavoprotein Subunit
SMT1	Sterol 24-C-Methyltransferase
SNU	U4/U6 Small Nuclear Ribonucleoprotein
SOD	Superoxide Dismutase
SQLE	Squalene Monooxygenase
sra	Stationary Phase Induced Ribosome-Associated Protein
TOA2	Transcription Initiation Factor TFIIA Small Subunit
trmB	tRNA (Guanine-N7)-Methyltransferase

TTC38	Tetratricopeptide Repeat Domain 38
UGP2	UDP-Glucose Pyrophosphorylase 2
UTP14	U3 Small Nucleolar Ribonucleoprotein
XPO1	Exportin-1
xynB	Xylanase
ZFP36L	Zinc Finger Protein 36 Homolog
α	20S Proteasome Alpha Subunit
β	20S Proteasome Beta Subunit
