# Supplementary material for: Multiomics Approach Reveals the Inhibitory Effects of Protocatechuic Acid on the Marine Dinoflagellate Scrippsiella acuminata
Source: Microorganisms. 2026 Mar 1;14(3):561. doi: 10.3390/microorganisms14030561 (PMC13028991; doi:10.3390/microorganisms14030561)
Supplement: Supplementary file 1 [file microorganisms-14-00561-s001.zip › Supplementary materials S2. Tables for methods.pdf]

Multiomics Approach Reveals the Inhibitory Effects of Protocatechuic Acid on the Marine Dinoflagellate *Scrippsiella acuminata*

Xin Zhang[1, †], Mei-yao He[2, †], Di Wang[1], Meimei Wang[1], Hongxin Liu[1], Jihui Wang[1], Shunshan Duan[2] and Meng Liu[1, *]

1 School of Life and Health Technology, Dongguan University of Technology, Dongguan 523808, China;

2 Department of Ecology, Jinan University, Guangzhou 510632, China.

* Corresponding author e-mail address: liumengpro2015@outlook.com (M. Liu)

† These authors contributed equally to this work.

Table S8 Liquid chromatography elution gradient of peptide fraction separation.

Time (min)	Flow rate (mL min^{-1})	A (%)	B (%)
0	1	97	3
10	1	95	5
40	1	80	20
68	1	60	40
70	1	30	70
74	1	0	100
78	1	0	100
80	1	0	100
90	1	0	100

Table S9 Elution gradient of liquid chromatography.

Time (min)	Flow rate (nL min^{-1})	A (%)	B (%)
0	600	94	6
2	600	83	17
82	600	60	40
84	600	45	55
85	600	0	100
90	600	0	100

Table S10 Elution gradient of liquid chromatography.

Time (min)	A%	B%
0	98	2
1.5	98	2
12	0	100
14	0	100
14.1	98	2
16	98	2
