# Supplementary material for: Multiomics Approach Reveals the Inhibitory Effects of Protocatechuic Acid on the Marine Dinoflagellate Scrippsiella acuminata
Source: Microorganisms. 2026 Mar 1;14(3):561. doi: 10.3390/microorganisms14030561 (PMC13028991; doi:10.3390/microorganisms14030561)
Supplement: Supplementary file 1 [file microorganisms-14-00561-s001.zip › Supplementary materials S4. Methods in detail.pdf]

Multiomics Approach Reveals the Inhibitory Effects of Protocatechuic Acid on the Marine Dinoflagellate *Scrippsiella acuminata*

Xin Zhang[1, †], Mei-yao He[2, †], Di Wang[1], Meimei Wang[1], Hongxin Liu[1], Jihui Wang[1], Shunshan Duan[2] and Meng Liu[1, *]

1 School of Life and Health Technology, Dongguan University of Technology, Dongguan 523808, China;

2 Department of Ecology, Jinan University, Guangzhou 510632, China.

* Corresponding author e-mail address: liumengpro2015@outlook.com (M. Liu)

† These authors contributed equally to this work.

1. Methods

1.1 Algal Culture and Experimental Design

S. acuminata (previously named as *Scrippsiella trochoidea*) (Dinoflagellata) was purchased from Shanghai Guangyu Biological Technology Co., Ltd. The algal strain was cultured in F/2 medium under static conditions in an artificial climate incubator (5401-CC275TL2H, Xutemp, Hangzhou) supplemented with artificial sea salt (Dragon King, Guangzhou) to achieve a salinity of 29±1 psu. The temperature was set to 20±1 °C, and the light intensity was set to 100 μmol $m^{-2}s^{-1}$. The light-dark cycle used was 12 h:12 h, with gentle shaking three times a day to ensure that the cells did not settle at the bottom.

S. acuminata in the exponential growth phase (with a cell density of approximately 50,000 cells/mL) were exposed to protocatechuic acid at the half-maximal inhibitory concentration (0.20 mM) for 48 h as the treatment group and labeled PA for 48 h. The control group consisted of *S. acuminata* cultured under normal conditions for 48 h and labeled the control for 48 hours. Both groups were duplicated at least three times for further analysis and measurement.

1.2 Transcriptomic Analysis

1.2.1 Extraction of RNA and library preparation

Each sample underwent a process in which 100 mL of the algal supernatant was removed and centrifuged at 3500 rpm for 6 min, after which the supernatant was discarded, and the sample was promptly frozen in liquid nitrogen. Additionally, each sample was filtered through a 5 μm filter membrane (Millipore, USA) to collect the algal cells, which were then rapidly frozen in liquid nitrogen and stored at -80 °C for RNA extraction.

For RNA extraction, a TRIzol reagent kit (Promega, USA) was used. Following extraction, RNA was dissolved in 200 μL of RNase-free water and stored at -80 °C. RNA purity was assessed via a NanoPhotometer spectrophotometer (Implen, Germany) to determine the OD260/280 and OD260/230 ratios, whereas RNA integrity was precisely evaluated via an Agilent 2100 bioanalyzer (Agilent, USA) to obtain the RNA integrity number (RIN).

Library construction was started with >1 μg of total RNA. mRNAs with polyA tails were enriched via oligo (dT) magnetic beads, followed by random fragmentation in NEB fragmentation buffer with divalent cations. The fragmented mRNA subsequently served as a template for synthesizing the first cDNA strand, followed by RNA chain degradation with RNase H and synthesis of the second cDNA strand via DNA polymerase I and dNTPs. The purified double-stranded cDNA was subjected to end repair, adenylation, and ligation of sequencing adapters. cDNAs of approximately 250-300 bp were selected via AMPure XP beads and subjected to PCR amplification. The PCR products were purified to obtain the library. Different libraries were pooled on the basis of the effective concentration and desired output data volume for Illumina sequencing, generating 150 bp paired-end reads.

1.2.2 De novo assembly and sequencing

The raw sequencing data were filtered to remove reads with adapters, reads containing N, and low-quality reads. The filtered clean reads were assembled via Trinity (v2.4.0), and transcript clustering and redundancy reduction were performed via Corset (v4.6). The unigene database was constructed from the longest sequence of each crown cluster.

1.2.3 Gene annotation and differential analysis

For comprehensive gene function analysis, gene function annotation was conducted against seven major databases: Nr, Nt, Pfam, KOG/COG, Swiss-Prot, KEGG, and GO. RSEM (v1.2.15) was used to map the clean reads to the unigenes, and DEGSeq (v1.12.0) was used for differential analysis of the unigenes.

The Benjamini and Hochberg method was used to adjust the P value, with padj < 0.05 and $|\log2foldchange| > 1$ indicating significant differential expression. Enrichment analysis of differentially expressed genes (DEGs) was performed via GOseq (v1.10.0) for GO database annotations and KOBAS (v2.0.12) for KEGG pathway enrichment analysis, both of which are based on hypergeometric distributions.

1.3 Proteomic Analysis

1.3.1 Proteome sample extraction

The sample preparation process involved filtering each sample through a 5 μm filter membrane (Millipore, USA) to collect algal cells, which were then rapidly frozen in liquid nitrogen and stored at -80 °C until subsequent protein extraction. Upon retrieval, the samples were ground into powder at low temperature and transferred to centrifuge tubes precooled in liquid nitrogen. An appropriate protein lysis mixture (100 mM ammonium bicarbonate, 6 M urea, 0.2% SDS; pH 8.0) was added to each sample, followed by shaking, mixing, and sonication in an ice-water bath for 5 min to ensure complete lysis. After centrifugation at 4 °C and 12000 × g for 15 min, the supernatant was collected, and 10 mM dithiothreitol (DTT) was added for the reaction at 56 °C for 1 h, followed by the addition of an adequate amount of iodoacetamide (IAM) for the reaction in the dark at room temperature for 1 h. The samples were then precipitated with four volumes of precooled acetone at -20 °C for at least 2 h, followed by centrifugation at 4 °C and 12000 × g for 15 min to collect the precipitate. The precipitate was resuspended, washed with 1 mL of precooled acetone at -20 °C, centrifuged again, air-dried, and dissolved in an appropriate amount of protein dissolution solution (6 M urea, 100 mM TEAB; pH 8.5).

1.3.2 Quality detection and TMT labeling

The standard protein solution of BSA was prepared according to the instructions of the Bradford protein quantification kit with a concentration gradient ranging from 0 to 0.5 μg/μL. BSA standard protein solutions with different concentration gradients and sample solutions with different dilution ratios were added to a 96-well plate, the volume was adjusted to 20 μL, and each gradient was repeated three times. The absorbance at 595 nm was determined by rapidly adding 180 μL of G250 dyeing solution and allowing it to stand at room temperature for 5 min. A standard curve was drawn with the absorbance of the standard protein mixture, and the protein concentration of the sample was calculated.

After concentration measurement, 20 μg protein samples were loaded onto 12% SDS-PAGE gels, with the stacking gel run at 80 V for 20 min and the separating gel run at 120 V for 90 min. After electrophoresis, the gel was stained with Coomassie Brilliant Blue R-250 and destained until the bands were visible. For protein digestion, 120 μg of protein sample was mixed with protein dissolution solution, trypsin, and TEAB buffer and then digested at 37 °C overnight. The digested samples were acidified with 1% formic acid, centrifuged, passed through a C18 desalting column, washed, and eluted. The eluted samples were combined and lyophilized, followed by dissolution in TEAB buffer and labeling with TMT reagent. The reaction was stopped with ammonia, and the labeled samples were mixed, desalted, and lyophilized.

1.3.3 Separation of peptide fractions and LC-MS/MS analysis

Mobile phases A (2% acetonitrile, adjusted pH to 10.0 using ammonium hydroxide) and B (98% acetonitrile, adjusted pH to 10.0 using ammonium hydroxide) were used to develop a gradient elution. The lyophilized powder was dissolved in solution A and centrifuged at 12,000 × g for 10 min at room temperature. The sample was fractionated via a C18 column (Waters BEH C18 4.6×250 mm, 5 μm) on a Rigol L3000 HPLC system, and the column oven was set at 50 °C. The details of the elution gradient are shown in Supplementary2, Table 1. The

eluates were monitored at UV 214 nm, collected in a tube per minute and finally combined into 10 fractions. All the fractions were dried under vacuum and then reconstituted in 0.1% (v/v) formic acid (FA) in water.

For transition library construction, shotgun proteomics analyses were performed via an EASY-nLCTM 1200 UHPLC system (Thermo Fisher, USA) coupled with a Q Exactive HF-X mass spectrometer (Thermo Fisher, USA) operating in data-dependent acquisition (DDA) mode. A 1 μg sample was injected into a custom-made C18 Nanotrap column (2 cm×75 μm, 3 μm). The peptides were separated on a custom-made analytical column (15 cm×150 μm, 1.9 μm) via linear gradient elution, as listed in Supplementary2, Table 2. The separated peptides were analyzed by a Q Exactive HF-X mass spectrometer (Thermo Fisher, USA) with an ion source of Nanospray Flex™ (ESI), a spray voltage of 2.3 kV and an ion transport capillary temperature of 320 °C. The full-scan range from m/z 350 to 1500 with a resolution of 60000 (at m/z 200), the automatic gain control (AGC) target value was 3×106, and the maximum ion injection time was 20 ms. The top 40 precursors with the highest abundance in the full scan were selected and fragmented by high-energy collisional dissociation (HCD) and analyzed via MS/MS, where the resolution was 15000 (at m/z 200) for 6 plexes (45000 for 10 plexes, m/z 200). The automatic gain control (AGC) target value was 5×104, the maximum ion injection time was 45 ms, the normalized collision energy was set as 32%, the intensity threshold was 1.9×10^{5}, and the dynamic exclusion parameter was 20 s.

1.3.4 Protein quantification, identification and functional analysis of DEPs

The raw data files were imported into Proteome Discoverer 2.2 software for database searching and peptide/protein quantification. The filtered search results were further refined on the basis of reliability criteria. To determine the significance of the difference, the relative quantitative value of each protein in the two comparison pairs was tested via a t test, and the corresponding P value was calculated as a significance indicator. When the P value was ≤0.05

and the FC was ≥1.5, the protein expression was upregulated. When the P value was ≤0.05 and the FC was ≤ 0.67, the protein expression was downregulated.

Gene Ontology (GO) and KEGG pathway enrichment analyses were subsequently conducted to identify functions and pathways significantly associated with the DEPs. Specifically, all DEPs were mapped to the terms in the Gene Ontology (GO) database to calculate the number of proteins for each term, and a hypergeometric test was subsequently applied to identify GO entries that were significantly enriched compared with the background of all proteins and within the differential proteins. The method for significant enrichment analysis of KEGG pathways is the same as that for GO functional enrichment analysis; KEGG pathways are used as the unit, and the hypergeometric test is applied to find pathways that are significantly enriched compared with the background of all identified proteins and within the differential proteins. After annotation and quantification of proteins with InterPro (IPR), the enrichment probability was calculated via hypergeometric distribution to obtain the enrichment of differential proteins in structural domains.

1.4 Metabolomic Analysis

1.4.1 Metabolite extraction

For metabolomic analysis, six biological replicates were collected for each experimental group. Algal cells and extracellular culture medium samples were filtered through a 5 μm filter membrane (Millipore, USA) and rapidly frozen in liquid nitrogen before being stored at -80 °C for metabolite extraction. The cell samples (100 mg) were mixed with 500 μL of 80% methanol solution containing 0.1% formic acid to precipitate proteins, followed by sonication for 6 min. After centrifugation at 15000 rpm and 4 °C for 10 min, the supernatant was collected, diluted with 60% methanol and mass spectrometry-grade water, and filtered through a 0.22 μm filter (15000 × g, 4 °C, 10 min) for LC-MS analysis. Extracellular culture medium samples (100 μL) were similarly treated with 400 μL of mass spectrometry-grade methanol. An equal volume of each experimental sample was combined to create a quality control (QC) sample for

analysis. Samples consisting of a 60% methanol solution containing 0.1% formic acid were used as blank samples.

1.4.2 Metabolite identification

Chromatographic analysis was conducted using a Hypersil Gold column (C18) (Thermo, USA) at 40 °C with a flow rate of 0.2 mL/min. A gradient elution gradient was used for chromatographic elution (Supplementary2, Table 3). The mobile phase for the positive mode consisted of 0.1% formic acid (A) and methanol (B), whereas for the negative mode, it consisted of 5 mM ammonium acetate (A) and methanol (B). Mass spectrometry scanning was performed in the m/z range of 100-1500, with the following ESI source settings: a spray voltage of 3.2 kV, sheath gas flow rate of 35 arb, aux gas flow rate of 10 arb, and capillary temperature of 320 °C. Both positive and negative ion modes were used, with data-dependent MS/MS scans.

The raw data files were imported into Compound Discoverer 3.0 (CD) for database searching and peak alignment. Peak extraction was performed based on set criteria, and compounds were identified via molecular and fragment ions compared against the mzCloud and Chemspider databases. Compounds with a coefficient of variance (CV) less than 30% in the QC samples were considered for final identification. The metabolites are screened during the identification process. The priority order for metabolite identification results is mzCloud > mzVault > ChemSpider. The identification results are also displayed in the supplementary materials.The blank sample was used to remove background ions, and the quantitative results were normalized to obtain qualitative and quantitative results.

The functional and classification annotations of the identified metabolites were performed via major databases, including KEGG, HMDB, and LIPID MAPS. By annotating the identified metabolites with these databases, one can understand the functional characteristics and classification of different metabolites.

1.4.3 Differentially abundant metabolite analysis

Partial least squares discrimination analysis (PLS-DA) was subsequently conducted to construct a model of the relationships between metabolite expression levels and sample categories. The PLS-DA models for each comparison group are evaluated via 7-fold cross-validation, and the model evaluation parameters (R^2, Q^2) are obtained. The closer R^2 and Q^2 are to 1, the more stable and reliable the model is. To determine the quality of the model, permutation validation is performed to check whether the model is "overfit". The absence of "overfitting" indicates that the model can describe the samples well and can be used as a premise for finding groups of model biomarkers, whereas "overfitting" suggests that the model is not suitable for describing the samples and should not be used for subsequent analysis. By randomly shuffling the group labels of each sample and then modeling and predicting, each modeling corresponds to a set of R^2 and Q^2 values. The regression lines of Q^2 and R^2 can be obtained via 200 shuffles and subsequent modeling. A reliable model's Q^2 should be significantly greater than the Q^2 obtained from shuffled group modeling, and when the R^2 data are greater than the Q^2 data and the Q^2 regression line intersects the Y-axis at less than 0, the model is not "overfitted".

The variable importance in the projection (VIP) values of the first principal component of the PLS-DA model are used, where VIP values represent the contribution rate of the differences in metabolites between groups; the fold change (FC) is the ratio of the mean of all biological replicate quantitative values of each metabolite in the comparison group; and combined with the P value of the t test to find differentially expressed metabolites, with thresholds set for VIP > 1.0, fold change (FC) > 2.0 or FC < 0.5, and P value < 0.05, to screen for differentially abundant metabolites.
